# Positional Isomers of B_6_C_6_N_6_ Nanorings: Stability, Reactivity, and Optical Properties from First Principles

**DOI:** 10.3390/nano16150953

**Published:** 2026-08-03

**Authors:** Xin Chen, Peipei Li, Shusheng Gong

**Affiliations:** 1 School of Materials and Chemical Engineering, West Anhui University, Lu’an 237012, China; cx810101@163.com (X.C.);; 2 Anhui Province Key Laboratory of Conservation and Utilization for Dabie Mountain Special Bio-Resources, West Anhui University, Lu’an 237012, China

**Keywords:** B_6_C_6_N_6_ cyclic nanorings, positional isomers, reactivity, aromaticity, UV–Vis absorption

## Abstract

The positional arrangement of BN and CC units in B_6_C_6_N_6_ cyclic nanorings profoundly influences their stability, electronic structure, optical response, and reactivity. Here, we comparatively investigate eight positional isomers (C1–C8) using DFT and TD-DFT calculations. Among C1–C8, C1 is the most stable, and C8 is the most unstable in the range of 200–1000 K. Their relative stability is governed by B-N charge separation, homonuclear B-B and N-N defects (charge repulsion), and bond-angle distortion (ring tension). The HOMO–LUMO gaps range from 4.40 eV (C3) to 8.45 eV (C2), indicating distinct kinetic stability. Aromaticity analysis reveals that all isomers are nonaromatic. In the gas phase, the lowest-energy absorption bands of C1 and C3 are located at about 429 nm and 606 nm, respectively. Due to different transition mechanisms, namely locally excited (LE) for the former and charge-transfer (CT) for the latter, solvent polarity has dramatically different influence on these two absorption bands. Compared to their positions in the gas phase, these absorption bands are blue-shifted about 20 nm and 220 nm in water, respectively. Reactivity analysis identifies the B-B bond in C7 as the strongest electrophilic site (LEAE = −2.93 eV), with the surrounding framework serving as nucleophilic domains, endowing C7 with the strongest bifunctional reactivity. This work establishes a comprehensive structure–property map for B_6_C_6_N_6_ isomers, providing guidance for designing BCN-based nanorings for catalysis, molecular recognition, and optoelectronics.

## 1. Introduction

Hexagonal boron nitride (h-BN) is a structural analogue of graphene, yet their electronic characters are dramatically different. h-BN has a wider band gap, polar B–N bonds, and distinct optical phonon modes [1,2]. These differences underpin the applications of h-BN in deep-ultraviolet emitters, dielectric layers, and thermal management and have also motivated the search for additional BN-based nanostructures with tailored properties [2,3]. By mixing BN and carbon domains, ternary B/C/N compounds may tune their band gaps and polarity, which in turn affect charge distribution, adsorption selectivity, and optical transitions [4].

Experimental and theoretical studies on zero-dimensional BN compounds (such as B_9_N_9_ and B_6_C_6_N_6_) and their analogues have attracted considerable interest, inspired in part by the first synthesis of cyclo[18]carbon (C_18_) in 1989 [5]. C_18_ has since been synthesized from different precursors, including C_18_Br_6_ and C_18_(CO)_6_ [6,7,8], and C_18_-related chemistry has been thoroughly reviewed [9]. In parallel, ring-like BN compounds have been incorporated into nanoresonators for optical gas sensing, demonstrating their device relevance [10]. However, while these experimental advances provide a stimulating backdrop, they do not imply that the mixed B_6_C_6_N_6_ ring is synthetically accessible or electronically similar to either C_18_ or B_9_N_9_; indeed, the synthesis of such ternary B/C/N nanorings may present substantial challenges. The present work therefore does not aim to propose synthetic routes but rather to establish a theoretical baseline for the structural and electronic properties of B_6_C_6_N_6_ isomers, which may inform future experimental efforts.

All of monocyclic C_18_, B_9_N_9_, B_6_C_6_N_6_, and B_2_N_2_C_14_ have 18 π electrons and are thus isoelectronic. Early DFT studies of B_9_N_9_ focused on its intrinsic stability and electronic structure [11,12]. From this baseline, research has branched into multiple application-oriented directions. For energy storage, B_9_N_9_ has been evaluated as a hydrogen storage medium [13] and as an anode component for magnesium-ion batteries [14]. For gas sensing, its sensitivity to small molecules such as CO, NO, NH_3_, SO_2_, and SF_6_ decomposition products has been systematically assessed, often with metal doping or Al substitution proposed to enhance selectivity [15,16,17]. In environmental chemistry, both pristine and Sc-decorated B_9_N_9_ rings have been shown to adsorb nitrobenzene effectively [18]. In the biomedical context, B_9_N_9_ has been investigated for detecting diabetes biomarkers (indole in exhaled breath) [19]; anticancer drug derivatives [20]; neurotransmitters, including dopamine and tyramine [21]; and small phenolics such as resorcinol [22]. The interaction between B_9_N_9_ and bio-relevant molecules has also been examined through quantum chemical methods [23]. These works collectively confirm that B_9_N_9_ can serve as a versatile prototype for evaluating the sensing, storage, and adsorption capabilities of inorganic ring systems.

Introducing carbon atoms into the BN framework gives rise to BCN hybrid rings, in which the distribution of B–N and C–C motifs can generate multiple positional isomers with distinct properties. Recent work on B_2_N_2_C_14_ has shown that isomers containing BNBN and BNNB units exhibit different relative energies and electronic structures [24,25]. Pioneering theoretical studies on BN-substituted cyclo[18]carbon analogues have established the fundamental stability trends for mono- and di-substituted systems with directly bonded B–N pairs [26], and more recently, the positional isomerism of mono-substituted BNC_16_ rings has been systematically explored [27]. The C_6h_-symmetric B_6_C_6_N_6_ ring, with its perfectly alternating (–B–C–N–)_6_ configuration, was found to be more aromatic than C_18_ and B_9_N_9_ because the carbon atoms can modulate the electron delocalization pattern [28]. Hybrid assemblies of B_6_C_6_N_6_ and B_9_N_9_ with superalkali M_3_O clusters exhibit enhanced nonlinear optical properties [29]. Notably, however, the alternating (–B–C–N–)_6_ structure has already been systematically characterized in Ref. [26]; the present work deliberately targets the unexplored regime beyond this perfect order. Specifically, this work focuses on the influence of homonuclear B–B and N–N defects and variations in carbon backbone connectivity on the structural and electronic properties.

Despite these contributions, a comprehensive comparative survey of the positional isomers of B_6_C_6_N_6_ remains absent. Specifically, it is not yet known how the relative arrangement of the BN and CC units affects (i) the thermodynamic stability hierarchy, (ii) the site-specific reactivity toward electrophiles or nucleophiles, and (iii) the UV–vis absorption characteristics. To fill this gap, we perform a comparative DFT and TD-DFT study on a set of eight representative positional isomers of B_6_C_6_N_6_, rationally designed to cover two key structural variables: carbon backbone topology (continuous C_6_ chain vs. discrete –CC– fragments) and homonuclear bonding patterns (B–B and N–N defects). The study covers their optimized geometries, relative energies, frontier orbital levels, aromaticity indices, reactivity descriptors, and UV–vis absorption spectra, with the aim of establishing a comprehensive structure–property map for this family of BCN nanorings.

## 2. Computational Details

Because previous benchmarks and calculation works [24,25,28,30,31,32] have confirmed that ωB97XD [33]/def2-TZVP [34] and TD-ωB97XD/def2-TZVP are suitable for the calculations of C_18_ and its BN or BCN analogues, all DFT and TD-DFT calculations were performed at these levels using Gaussian 16 (Revision C.02) [35]. For geometry optimization, default convergence tolerances and SCF = Tight criteria were employed.

Vibrational frequency calculations were performed at the same level to confirm that all optimized structures are local minima (no imaginary frequencies). Thermodynamic quantities (G, S, C_V_, C_P_) were derived from the frequency output files using the Shermo program [36] under the ideal gas assumption at 298.15 K and 1 atm, with zero-point vibrational energies scaled by 0.9596. The quasi-RRHO model [37] was employed for low-frequency modes with an interpolation threshold of 100 cm^−1^. All reported relative energies (ΔE) and relative Gibbs free energies (ΔG) at 298.15 K are normalized to the most stable isomer C1. Temperature-dependent ΔG values (200–1000 K) are defined as ΔG_i_(T) = G_i_(T) − G_C1_(298.15 K), where G_C1_(298.15 K) is the absolute Gibbs free energy of C1 at 298.15 K, serving as a fixed reference point for all temperatures.

For UV–vis absorption spectra, the lowest 20 singlet excited states were calculated at the TD-ωB97XD/def2-TZVP level, with a full width at half-maximum of 0.15 eV for spectral simulation. Solvent effects were modeled using the SMD continuum solvation model [38].

Reactivity was evaluated using the orbital-weighted dual descriptor (Δf_w_) [39] and local electron attachment energy (LEAE) [40]. Aromaticity was analyzed via ELF-π and IRI-π [41]. All wavefunction analyses, including hole–electron analyses [42] and spectral simulations, were performed using Multiwfn 3.8 [43,44].

## 3. Results and Discussions

### 3.1. Geometrical Structures of Eight B_6_C_6_N_6_ Isomers

The eight cyclic B_6_C_6_N_6_ isomers examined in this work were not intended to exhaust all possible constitutional isomers. Instead, they were deliberately designed around two essential structural determinants: (i) carbon backbone topology: whether the six carbon atoms form a continuous polyene chain (C1, C3, C4) or are partitioned into three discrete –CC– fragments separated by BN-based bridging units (C2, C5–C8); (ii) homonuclear bond character: the presence and distribution of B–B and N–N bonds, which introduce intramolecular electrostatic repulsion and geometric strain. C1 and C2 are free of any homonuclear bonds; C5 contains only B–B bonds. C3, C4, C6, C7, and C8 contain N–N bonds to varying extents, with C8 bearing the highest number of N–N contacts; C4, C6, and C7 contain both B-B and N-N bonds.

Using this two-variable design matrix, we can disentangle the separate impacts of carbon connectivity and homonuclear bonding on the overall stability and geometric distortion of the B_6_C_6_N_6_ ring. As noted in the Introduction, the perfectly alternating (–B–C–N–)_6_ structure [26] is excluded to avoid redundancy. Furthermore, unstable isomers containing three or more consecutive N atoms are not considered as representative members of the main series [45,46].

**Figure 1:**
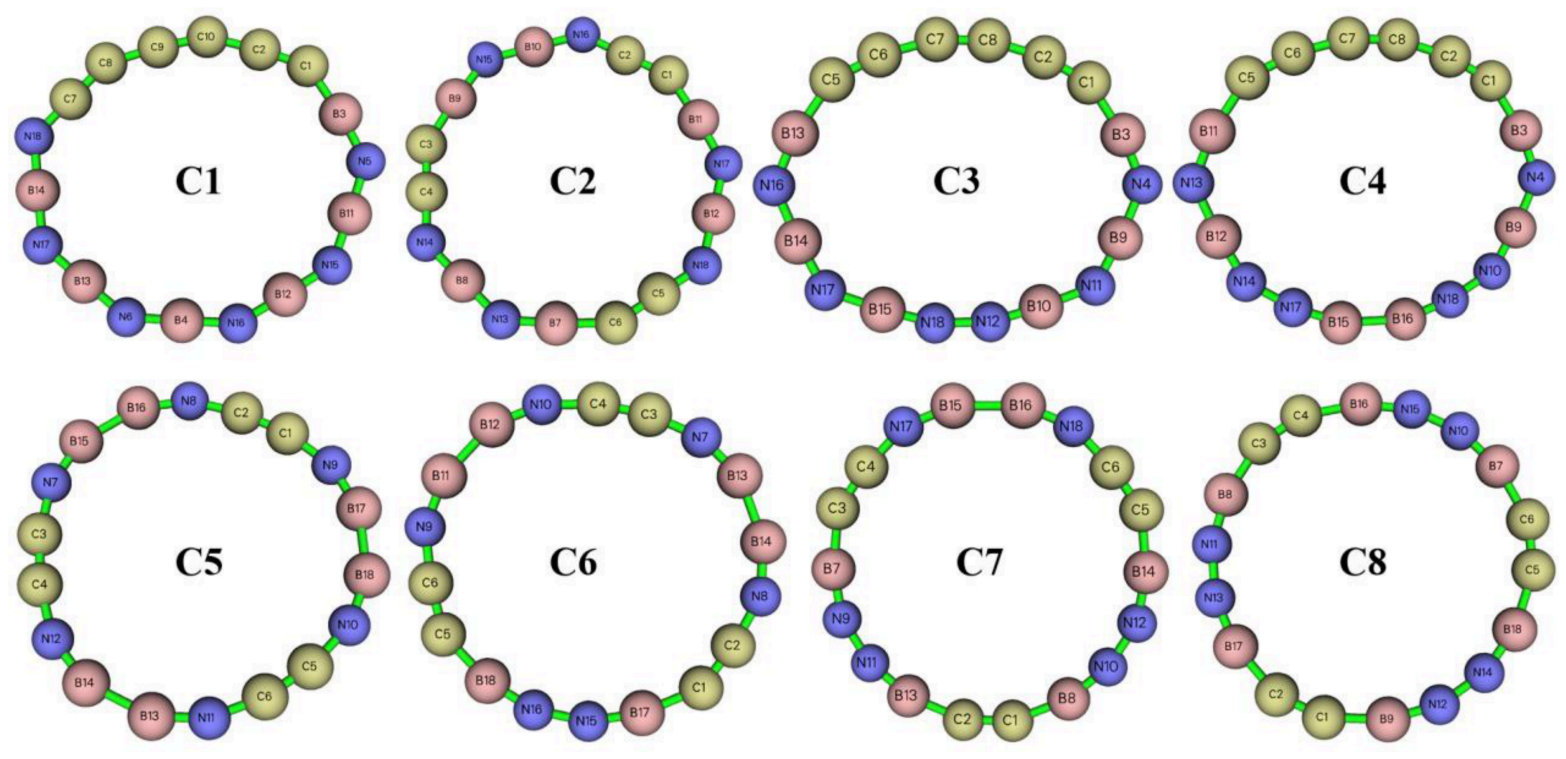
Structures of eight B_6_C_6_N_6_ compounds (C1–C8). The atoms in blue, yellow, and orange are N, C, and B atoms, respectively.

Eight cyclic B_6_C_6_N_6_ isomers adopt two distinct carbon backbone arrangements: C1, C3, and C4 contain an unbroken six-carbon chain in the ring, whereas C2, C5–C8 split six carbons into three discrete –CC– segments separated by diverse BN-based bridging units. Marked differences in homonuclear B–B and N–N bonds exist among these cyclic frameworks (Figure 1). C1 and C2 have no homonuclear B–B or N–N bonds; N–N bonds are present in C3, C4, C6, C7, and C8 (C8 possesses the largest number of N–N contacts), while B–B bonds occur in C4, C5, C6, and C7. Notably, C5 solely holds three B-B bonds and contains no N–N bonds. Homonuclear bonds introduce extra intramolecular repulsive forces, which aggravate geometric distortions at boron and nitrogen atomic centers. Such structural deformations can be quantitatively characterized by central bond angles and further correlated with the relative thermodynamic stability of each isomer. The optimized Cartesian coordinates of C1–C8 are shown in Table S1.

To comparatively quantify ring geometric distortion, we statistically analyzed B-centered bond angles (N–B–N, C–B–N, N–B–B) and N-centered bond angles (B–N–N, B–N–B, B–N–C) of all eight isomers, which directly reflect the local hybridization characteristics of B and N under different atomic coordination environments. By comparison, the C–C–C bond angle of all-carbon C_18_ is approximately 160°, while the bond angle of N–B–N and B–N–B in pure B_9_N_9_ rings are 179.3° and 140.7°, respectively. For the ideal C_6h_-symmetric defect-free B_6_C_6_N_6_ framework, three core heteronuclear bond angles (B–C–N, C–N–B, N–B–C) all converge near 160° (159.3°, 162.1°, 158.6°), representing the carbon-mediated homogenized geometric preference of the ternary B–C–N ring system [26]. The full statistical data of B-centered and N-centered bond angles for C1–C8 are summarized in Tables S2 and S3. Simulated IR spectra of compounds C1–C8 at gas phase is shown in Figure S1, C2 has much stronger B-N stretching vibration than others.

### 3.2. Thermodynamic Stability of the Eight B_6_C_6_N_6_ Isomers

Table 1 summarizes the thermodynamic parameters of eight B_6_C_6_N_6_ isomers at 298.15 K. The ranking of relative Gibbs free energies (ΔG) exactly matches that of relative electronic energies (ΔE) and average binding energies (average E_bind_, Table S4): C1 < C2 < C3 < C4 < C5 < C6 < C7 < C8. This consistent ordering confirms that electronic energy governs the room-temperature stability hierarchy, with thermal corrections exerting only secondary influences.

The calculations of E_bind_ and average E_bind_ are based on the following formula:

E_bind_ = E_total_(B_6_C_6_N_6_) − [6E(B) + 6E(C) + 6E(N)]

average E_bind_ = (E_total_(B_6_C_6_N_6_) − [6E(B) + 6E(C) + 6E(N)])/18

where E_total_(B_6_C_6_N_6_) is the ZPE (Zero-point energy)-corrected total energy of each B_6_C_6_N_6_ isomers; E(B), E(C), and E(N) are the ZPE-corrected energies of B, C, and N atoms calculated at ωB97XD/def2-TZVP level; and 0.9596 is used as the ZPE scaling factor.

For all isomers, ΔG is marginally higher than ΔE, owing to combined zero-point vibrational, thermal enthalpy and entropic corrections. Representative disparities include 0.49 kcal/mol for C2 (ΔE = 29.63 kcal/mol, ΔG = 30.16 kcal/mol), 1.27 kcal/mol for C5 (ΔE = 210.68 kcal/mol, ΔG = 212.20 kcal/mol), and 0.96 kcal/mol for C8 (ΔE = 237.54 kcal/mol, ΔG = 238.89 kcal/mol).

Entropies (S) span 123.17–127.42 cal/(mol·K), and the low-symmetry C1 possesses the largest entropy (127.42 cal/(mol·K)), while the D_3h_-symmetric C5 exhibits the smallest entropy (123.17 cal/(mol·K)). The vibrational heat capacities show limited variation across the eight structures: C_V_ ranges from 56.19 to 60.12 cal/(mol·K), and C_P_ ranges from 58.18 to 62.10 cal/(mol·K). Such minor deviations fail to alter the relative stability sequence at 298.15 K. C1 adopts C_s_ symmetry; C2 adopts C_3h_ symmetry. C5 and C8 belong to D_3h_; C3, C4, C6, and C7 adopt C_2v_ symmetry.

**Figure 2:**
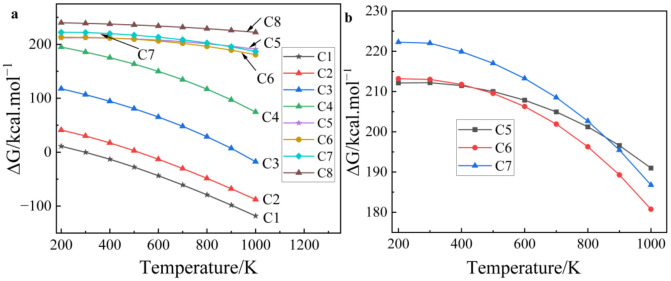
Gibbs free energy changes at different temperatures (200–1000 K) of C1–C8 (**a**) and C5–C7 (**b**).

Table S5 and Figure 2 present temperature-dependent relative Gibbs free energies spanning 200–1000 K, with G_C1_(298.15 K) serving as a fixed reference point for all temperatures. The absolute Gibbs free energies of all isomers decrease monotonically with increasing temperature, reflecting the increasing entropic contribution (−TΔS) at higher temperatures. At 500 K, C2 lies 30.44 kcal/mol higher in free energy than C1 (ΔG = −27.54 kcal/mol for C1 versus +2.90 kcal/mol for C2). The free energy gap between them remains nearly constant with increasing temperature, reaching 30.47 kcal/mol at 600 K (C1: −43.51 kcal/mol; C2: −13.04 kcal/mol) and 30.50 kcal/mol at 1000 K (C1: −118.22 kcal/mol; C2: −87.72 kcal/mol).

All isomers display monotonically declining ΔG values with rising temperature. For instance, the relative free energy of C8 decreases from 240.02 kcal/mol (200 K) to 222.37 kcal/mol (1000 K). The free energy separation between C1 and C8 slightly increases from 228.98 kcal/mol at 200 K to 263.50 kcal/mol at 500 K and then expands dramatically to 340.59 kcal/mol at 1000 K.

Two core conclusions can be drawn from the thermodynamic datasets. First, electronic energy dictates the room-temperature stability order, as small entropic corrections cannot reverse the energy hierarchy at 298.15 K. Second, thermal entropy effects dominate stability rankings above 500 K. The higher symmetry and distinct vibrational entropy of C2 render it thermodynamically superior to C1 starting at 600 K. Overall, the relative stability of B_6_C_6_N_6_ isomers is strongly temperature-dependent, while electronic factors remain dominant under ambient conditions.

**Figure 3:**
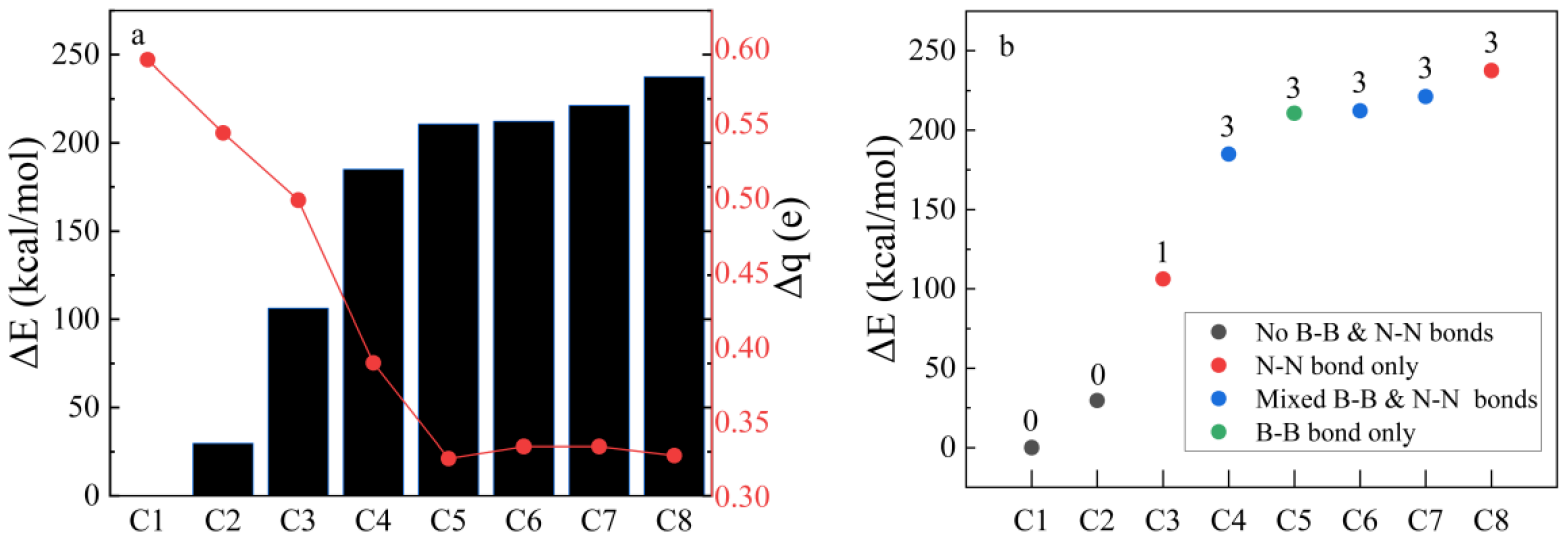
(**a**) The relative energy ΔE (black columns) and charge transfer Δq (red line with symbols) of C1–C8 isomers. (**b**) The relative energy ΔE distribution of each isomer. Numbers above scatter points represent the total number of homonuclear bonds in the B_6_C_6_N_6_ ring.

To identify the structural factors governing the stability hierarchy at the electronic level, we correlate the relative energies (ΔE) with two key descriptors: the B-N charge separation (Δq, Table 2) and the number of homonuclear defects. As shown in Figure 3a, ΔE exhibits a nearly monotonic inverse correlation with Δq: isomers with larger charge separation (C1, C2) are consistently more stable than those with smaller Δq (C5–C8). This trend confirms that the electrostatic attraction between Bδ^+^ and Nδ^−^ centers serves as the primary thermodynamic driving force.

However, Figure 3b reveals that the correlation is not strictly linear with the number of homonuclear bonds. For instance, C5 (three B–B bonds, Δq = 0.325 e) and C8 (three N–N bonds, Δq = 0.327 e) have nearly identical Δq values, yet C8 lies 27 kcal/mol higher in energy. This disparity unambiguously demonstrates that the type of homonuclear defect matters more than the defect count alone—N–N defects impose a larger energy penalty than B–B defects, consistent with the stronger lone-pair repulsion between adjacent Nδ^−^ centers. Similarly, C6 (two B–B + one N–N) is 9 kcal/mol more stable than C7 (one B–B + two N–N), further supporting the greater destabilizing effect of N–N adjacencies.

The overall stability hierarchy is therefore governed by a hierarchy of factors: (i) B–N charge separation as the primary descriptor, (ii) the type and number of homonuclear defects as the major penalty, and (iii) carbon-chain conjugation as a secondary compensatory effect. This multi-factor nature explains why a simple linear correlation with a single parameter is not observed—a point that itself underscores the complexity of the B_6_C_6_N_6_ potential energy surface.

Three factors collectively govern this ordering, operating hierarchically with diminishing weights.

B–N charge separation (Δq) serves as the primary descriptor. Δq is the difference between the average Hirshfeld charge of B atoms and that of N atoms for each isomer (Table 2 and Table S6a). C1 has the largest Δq (0.592 e) and the lowest energy (0 kcal/mol). C8 has the smallest Δq (0.327 e) and the highest energy (237.54 kcal/mol). From C1 to C8, Δq decreases monotonically while energy increases. In the C1–C4 range, Δq drops from 0.592 to 0.389 e, and energy rises from 0 to 184.93 kcal/mol. In the C5-C8 range, Δq values cluster narrowly (0.325–0.333 e), yet energy still climbs from 210.68 to 237.54 kcal/mol, indicating that secondary factors dominate when the primary factor is similar.

To verify the robustness of this descriptor, we also computed ADCH (atomic dipole corrected Hirshfeld) charges, which account for atomic dipole contributions and often provide improved charge distributions for polar systems. As summarized in Table S6b, the ADCH-derived Δq values follow the same monotonic trend as the Hirshfeld charges (Table S6a), confirming that the observed structure-stability correlation is not an artifact of the chosen population analysis scheme.

Homonuclear defects (B–B and N–N adjacencies) impose the major energy penalty. Three trends support this conclusion. First, the number of defects correlates with energy: C1 and C2 (zero defects) have energies below 30 kcal/mol, C3 (one defect) has 106.19 kcal/mol, C4–C7 (two or three defects) range from 184.93 to 221.29 kcal/mol, and C8 (three defects) has 237.54 kcal/mol. Second, N–N defects are more destabilizing than B–B defects. C5 (three B–B) and C8 (three N–N) have nearly identical Δq values (0.325 vs 0.327 e), but C8 is 26.86 kcal/mol higher in energy. The same trend appears in C6 vs C7: both have Δq = 0.333 e, but C6 (two B–B + one N–N) is 9.06 kcal/mol lower than C7 (one B–B + two N–N). Third, B-B defects compress B-centered bond angles more severely than N–N defects. The N–B–B angles (155–159°) are consistently lower than the C–B–N angles in N–N-containing isomers (162–168°), reflecting direct electrostatic repulsion between adjacent Bδ^+^ centers.

Bond-angle distortion provides direct geometric evidence for the strain imposed by defects. Defect-free isomers C1 and C2 maintain near-ideal N–B–N angles close to 180° (deviation < 5°), preserving near-sp hybridization of boron. When N–N defects are introduced, B–N–N angles deviate from the ideal sp2 value of 120° by 37–49°, reflecting lone-pair repulsion between adjacent Nδ^−^ centers. However, the correlation is not strictly linear: C8 shows moderate B-angle deviation (17.36°) but the highest energy, indicating that cumulative penalties from multiple N–N defects and six B–C bonds outweigh single-angle distortion. C4 shows the most extreme N-centered distortion (169.2°, deviation 49.2°) but lower energy than C7 and C8 because its continuous –C6– chain provides π- delocalization compensation.

The continuous carbon chain provides stabilization through π delocalization. C3 (continuous chain, one N–N) is 106.04 kcal/mol lower than C6 (discrete –CC– segments, two B–B + one N–N). C4 (continuous chain, one B–B + two N–N) is 36.36 kcal/mol lower than C7 (discrete –CC– segments, same defects). The compensation decreases as defect density increases because homonuclear defects disrupt the conjugation pathway.

In summary, the stability of B_6_C_6_N_6_ isomers is governed by a hierarchy of factors: B–N charge separation as the primary determinant, homonuclear defects as the major penalty, bond-angle distortion as geometric evidence of strain, and carbon-chain conjugation as a compensatory effect. These four factors operate simultaneously to produce the stability sequence. C1 combines the most favorable attributes (largest Δq, no defects, continuous carbon chain) and is therefore the global minimum. C8 combines the most severe features (three N–N defects, six B–C bonds) and is therefore the highest in energy.

### 3.3. Electronic Structures of Eight B_6_C_6_N_6_ Isomers

The HOMO and LUMO isosurfaces and TDOS (total density-of-state) and PDOS (partial density-of-state) plots of other compounds are listed in Figures S2 and S3. As seen in Figure S2, the HOMO and LUMO isosurfaces of C1 exhibit π_out_ character (i.e., perpendicular to the molecular plane) and are predominantly localized on the -C6- moiety, protruding toward the heteroatoms on their respective opposite sides. TDOS and PDOS plots of C1 indicate that orbitals of C atoms dominate the HOMO and LUMO isosurfaces (Figure S3). In C3 and C4, the HOMO shifts to the heteroatoms (π_out_), while the LUMO remains on the -C6- moiety (π_in_, i.e., parallel to the molecular plane). For C3 and C4, the main contribution to the HOMO arises from N and B, while the LUMO is almost exclusively dominated by C-atom orbitals. Among all compounds, C3 exhibits the smallest HOMO-LUMO gap (4.40 eV) and the highest-lying HOMO (−6.92 eV), while C2 shows the largest gap (8.45 eV) and the lowest-lying HOMO (−8.93 eV). Notably, the energy gaps of C3 and C4 (4.40 and 5.50 eV, respectively) are significantly smaller than those of the other compounds (6.50–8.45 eV) (Table 3).

### 3.4. Aromaticities of Eight B_6_C_6_N_6_ Isomers

The IRI-π (Interaction Region Indicator for π electrons) diagrams of C1–C8 (Figure S4) reveal circular isosurfaces around each chemical bond, indicating that all bonds possess both π_in_ and π_out_ interaction characteristics to varying strengths. The LOL-π (Localized Orbital Locator for π electrons) isosurfaces [47] further show that only C1 and C2 exhibit uninterrupted LOL-π_out_ isosurfaces, whereas all other LOL-π_in_ and LOL-π_out_ isosurfaces are discontinuous (Figure S5). This observation suggests that C1 and C2 may possess π_out_ aromaticity. Quantitative support for this assignment comes from the ELF-π isosurface isovalues [48,49] summarized in Table 4. For C1 and C2, the isovalues for ELF-π_out_ (0.625 and 0.655, respectively) are substantially larger than those for the other compounds (0.235–0.305) (Figure S6). Similarly, the isovalues for ELF-π_in_ and ELF-π in C1 and C2 are also notably higher than those in C3–C8. These results indicate that C1 and C2 are more aromatic and thus more stable than C3–C8. However, the aromaticity of C3–C8 does not correlate well with their relative stabilities, implying that aromaticity is not the sole factor governing their stability. For both ELF-π_out_ and ELF-π_in_, the values for compounds C1–C8 are lower than those of C_6h_-B_6_C_6_N_6_ (0.83 for π_out_ vs. 0.73 for π_in_) and D_9h_-B_9_N_9_ (0.78 vs. 0.56) [28]. Since D_9h_-B_9_N_9_ has been confirmed to have no aromaticity [28], all of the compounds C1-C8 are likewise considered nonaromatic.

### 3.5. UV–Vis Spectra of Eight B_6_C_6_N_6_ Isomers

Excited energy, excited wavelength, oscillator strength, and main molecular orbital transitions of the first 20 excited states of C1–C8 in gas phase are listed in Table S7. The most notable feature in Table 5 is State 4 of C3 (*f* = 0.114, λ = 543 nm), whose visible-light absorption makes it a promising candidate for bioimaging, optoelectronics, and photo-responsive materials. In contrast, absorption of C4, C6, and C7 are confined to the near- to deep-UV (283–374 nm), and C1 and C2 show only weak or moderate bright-state absorption in the visible/deep-UV regions.

**Figure 4:**
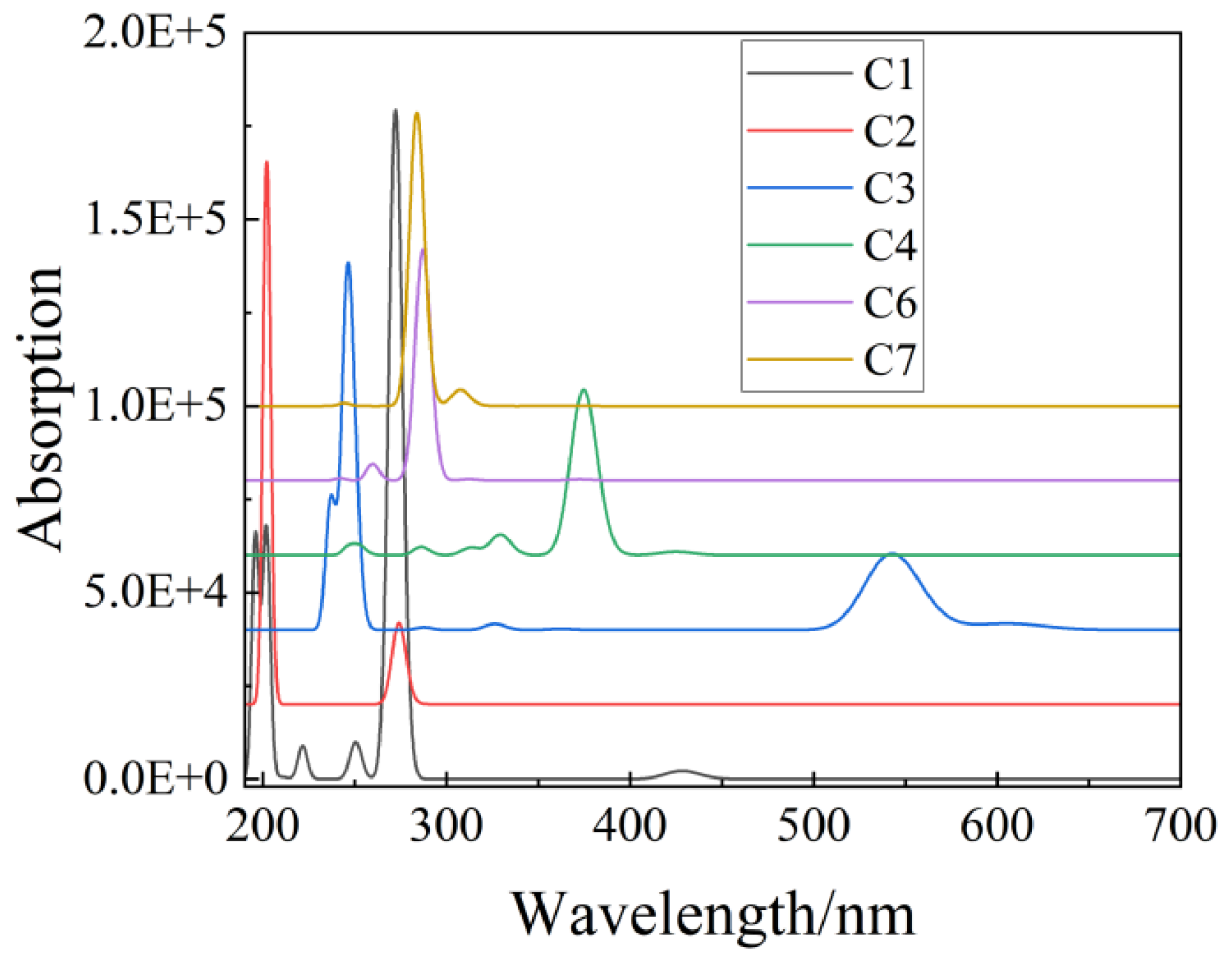
Simulated UV–Vis absorption spectra of compounds C1–C4 and C6–C7 in the gas phase.

For the entire series, the oscillator strengths provide a practical criterion for assessing utility. Values of *f* ≥ 0.1 (e.g., State 4 of C1, *f* = 0.997) indicate intense absorption suitable for fingerprinting and quantitation; 0.01 ≤ *f* < 0.1 (e.g., States 6/7 of C2) provides auxiliary structural information; and *f* < 0.01 (e.g., State 2 of C3) offers only theoretical insight into transition mechanisms. C5 and C8, with *f*_max_ < 0.01 across the first 20 excited states, are not viable for light harvesting (Table S6). Simulated UV–Vis spectra of compounds C1–C4 and C6–C7 at the gas phase (FWHM = 0.15 eV) are shown in Figure 4.

The hole–electron descriptors for C1–C8 are collected in Table 6, and the corresponding isosurfaces are shown in Figure S7. These data allow for the excited states to be classified as locally excited (LE) or charge-transfer (CT), as discussed below.

For C1, both S2 and S4 exhibit LE character, as evidenced by their relatively large Sr values (0.66 and 0.73 a.u.), short t distances (−1.31 and −1.38 Å), and high Ecoul values (5.41 and 4.52 eV). These quantitative features indicate strong hole–electron overlap and pronounced Coulomb attraction. This assignment is visually corroborated by the compact Sr isosurfaces and the substantial spatial overlap between hole and electron distributions observed in Figure S7. Similarly, C2 (S6 and S7) displays LE character, with even larger Sr values (0.75 a.u. for both), the shortest t distances (0.35 and −1.78 Å), and Ecoul values of 4.39 and 4.46 eV, reflecting the most confined excitations among all investigated states. The LE nature of C6 (S12) and C7 (S10) is also supported by their moderate-to-large Sr values (0.56 and 0.75 a.u.), relatively short t distances (1.82 and −1.23 Å), and Ecoul values of 3.38 and 4.02 eV. For all LE states, the HDI and EDI values are consistently high (HDI: 5.62–8.14; EDI: 4.26–7.23), indicating strong electron–hole interactions and efficient exciton binding.

In marked contrast, C3 (S2 and S4), C4 (S6), and C7 (S11) exhibit clear CT character, characterized by substantially smaller Sr values (0.28–0.52 a.u.), much larger t distances (2.30–5.18 Å), and lower Ecoul values (2.63–3.25 eV). Among these, C3 S4 shows the most pronounced CT feature, with the smallest Sr (0.28 a.u.) and the largest t (5.18 Å), indicating nearly complete spatial separation of the hole and electron. The CT assignment is visually confirmed by the dispersed Sr isosurfaces and the clearly separated hole and electron lobes in Figure S8. The HDI and EDI values of CT states are generally lower (HDI: 5.62–7.71; EDI: 4.26–5.89) than those of LE states, consistent with weaker electron–hole interactions arising from larger spatial separation.

It is noteworthy that C7 possesses both an LE state (S10) and a CT state (S11), depending on the excitation energy, indicating that a single compound can give rise to excited states of distinct natures. This duality underscores the importance of wavelength-selective excitation for targeted applications.

Overall, the quantitative descriptors in Table 6 and the visualized isosurfaces in Figure S6 are fully consistent with each other, showing no conflicting cases. The clear separation of Sr, t, and Ecoul values between LE and CT states-with LE states characterized by Sr ≥ 0.56 a.u., t ≤ 1.82 Å, and E_coul_ ≥ 3.38 eV and CT states by Sr ≤ 0.52 a.u., t ≥ 2.30 Å, and E_coul_ ≤ 3.25 eV-provides a robust and unambiguous criterion for distinguishing the two types of excitations in this series.

To investigate the influence of solvent effects on C1 and C3, structure optimization and frequency calculation under SMD solvent model were performed for them at the ωB97XD/def2-TZVP level, and their UV–Vis spectra were calculated at TD-ωB97XD/def2-TZVP level in three solvents (cyclohexane, acetonitrile, and water). No imaginary frequency was found under all above-mentioned conditions, and the simulated IR spectra of compound C1 and C3 in cyclo-hexane, acetonitrile, and water are shown in Figure S8. Table 7 summarizes the computed excitation properties of C1 and C3 at the gas phase and in three solvents of increasing polarity (cyclohexane, acetonitrile, and water).

**Figure 5:**
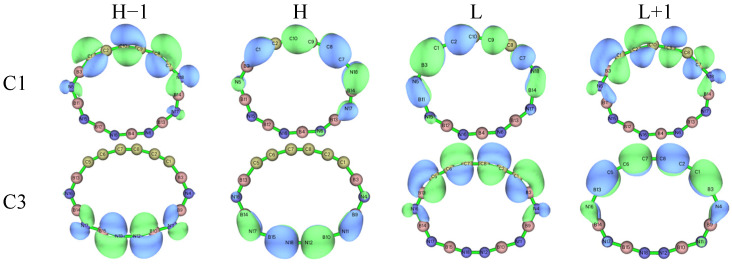
Isosurfaces of the involved frontier molecular orbitals (FMOs) for C1 and C3 (isovalue = 0.05 a.u.) at gas phase (Their FMO isosurfaces in the three solvents are like those at gas phase).

For C1 in the gas phase, cyclohexane, acetonitrile, and water, all lowest-energy electronic transitions with *f* > 0.1 include S2, while S4 consistently exhibits the largest oscillator strength (Table 7 and Table S8). Notably, the *f* values of both excited states increase with increasing solvent polarity. As the solvent polarity increases, the longest absorption wavelength undergoes a slight blue shift (from 428.51 nm in the gas phase to 423.76 nm in cyclohexane, 409.19 nm in acetonitrile, and 407.55 nm in water), whereas λ_max_ remains almost constant at approximately 273 nm. These observations indicate that solvent polarity has little influence on the UV–Vis spectra of C1 (Figure S9). For C1 in all four media, both S2 and S4 arise mainly from HOMO→LUMO (π_out_→π_out_*) and HOMO−1→LUMO+1 (π_in_→π_in_*) transitions. Since the isosurfaces of the four relevant FMOs (HOMO−1, HOMO, LUMO, and LUMO+1) are localized on the –C6– fragment with slight extension to both adjacent sides (Figure 5), both S2 and S4 can be assigned as locally excited (LE) states, as can also be inferred from the data in Table S6.

In comparison, C3 exhibits markedly different spectral features, and the corresponding transition state of the two lowest-energy absorption bands vary according to solvent polarity. At the gas phase, the contributions of HOMO→LUMO+1 (π_out_→π_out_*) and HOMO-1→LUMO (π_in_→π_in_*) correspond to both lowest-energy absorption bands (Table 7 and Table S9). Along the increase in solvent polarity, the first lowest-energy absorption bands gradually are contributed solely by HOMO→LUMO+1 (πout→πout*), and the second lowest-energy absorption bands are gradually dominated by HOMO−1→ LUMO (π_in_→π_in_*). Figure 5 and Table S7 jointly confirm that the two lowest-energy absorption bands of C3 in the four media are all characterized by intramolecular CT. Meanwhile, the two lowest-energy absorption band shifts dramatically to shorter wavelengths: 605.78 nm (gas phase)→492.24 nm (cyclohexane)→380.93 nm (acetonitrile)→377.80 nm (water), corresponding to a total blue shift of more than 220 nm.

In summary, C1 and C3 display fundamentally different solvatochromic behaviors, stemming from their distinct orbital distributions and electronic transition modes. LE-type excitation in C1 produces little change in dipole moment, whereas CT-type excitation in C3 leads to a large dipole-moment reduction upon photoexcitation.

### 3.6. Reactivities of Eight B_6_C_6_N_6_ Isomers

**Figure 6:**
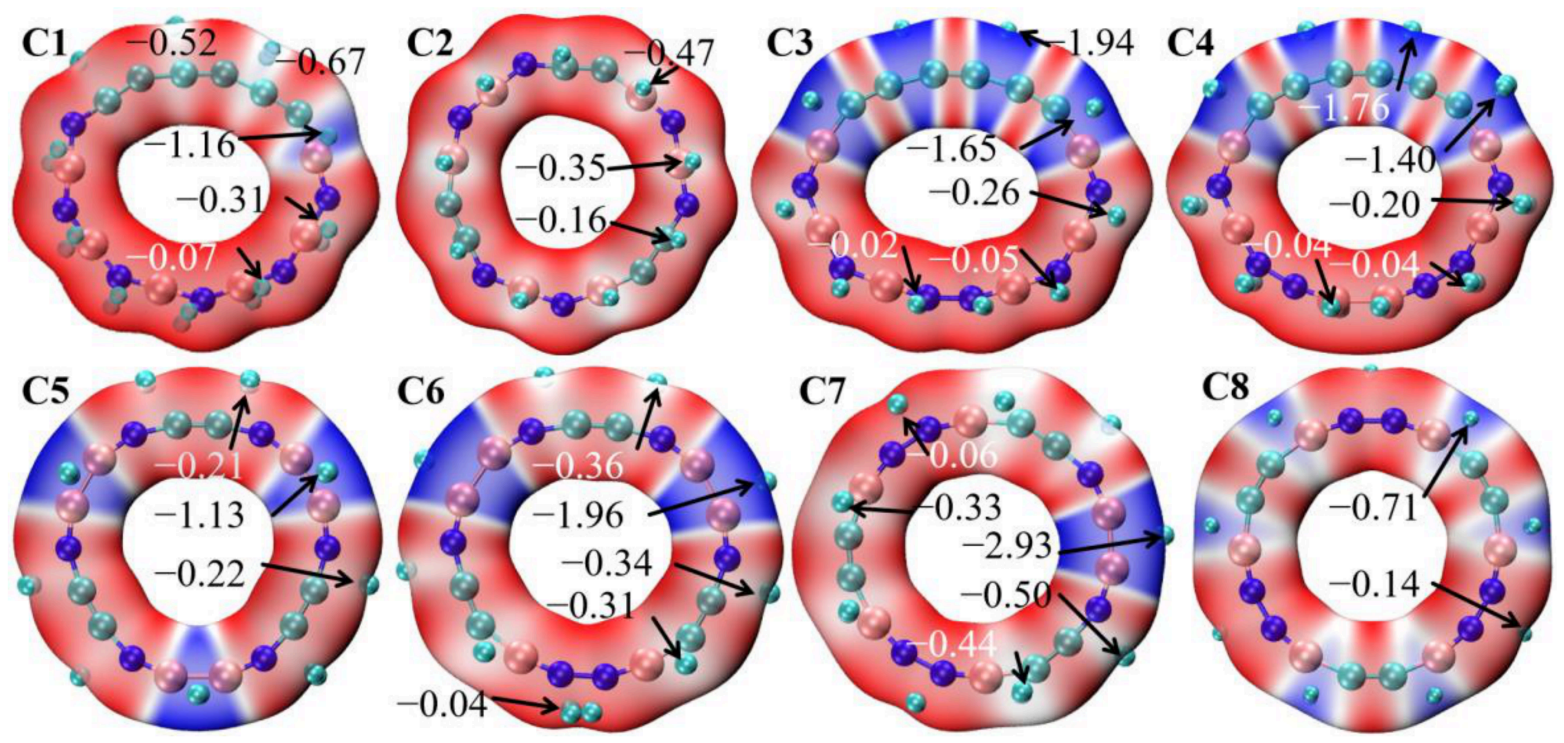
LEAE maps with labeled LEAE values (in eV) at each surface minimum point (isovalue = 0.004 a.u.).

The local atomic electron energy (LEAE) maps (Figure 6), orbital-weighted dual descriptor (OWDD) isosurfaces (Figure S10), and color-filled maps of OWDD (Figure S11) jointly reveal the reactive sites of C1–C8.

In C1, the most negative LEAE (−1.16 eV) coincides with the greenest OWDD lobe at the B atom adjacent to the –C6– fragment, identifying this B atom as the primary electrophilic site (susceptible to nucleophilic attack). In C7, the B–B bond exhibits both the deepest blue LEAE region (−2.93 eV) and the most pronounced green OWDD lobe, while the rest of the ring periphery displays red LEAE and blue lobes. This unambiguously assigns the B–B bond as the electrophilic center and the surrounding framework as nucleophilic domains. The electron-deficient B–B bond (strong electrophilic site) could serve as an anchoring point for nucleophilic substrates or catalysts, while the electron-rich framework (nucleophilic domains) may facilitate cooperative interactions with electrophilic species. Such dual functionality is particularly attractive for bifunctional catalysis, where spatially separated acidic and basic sites within a single molecule can cooperatively activate substrates in a synergistic manner. Furthermore, the well-defined charge separation in C7, combined with its cyclic nanoring structure, may enable selective molecular recognition, host–guest chemistry, or self-assembly on polar surfaces. The extreme electron polarization also suggests C7 as a promising candidate for nonlinear optical materials, where large intramolecular charge transfer enhances the optical response. In contrast, C2 and C8 show much less negative extreme LEAE (−0.47 and −0.71 eV, respectively) and contracted lobes, indicating their weakened dual reactivity relative to C7.

## 4. Conclusions

In this work, we have comparatively investigated eight positional isomers of B_6_C_6_N_6_ cyclic nanorings (C1–C8) using DFT and TD-DFT calculations, focusing on their stability, electronic structure, aromaticity, UV–Vis absorption, solvatochromic behavior, and reactivity.

The relative stability follows the order C1 > C2 > C3 > C4 > C5 > C6 > C7 > C8, and their relative stability is governed by B–N charge separation, homonuclear B–B and N–N defects, and bond-angle distortion. Continuous carbon chains can provide partial stabilization through π-delocalization. Aromaticity analysis indicates that C1 and C2 possess relatively stronger π_out_ electron delocalization, yet all eight isomers are nonaromatic.

UV–Vis absorption varies significantly across the series. In the gas phase, the first 20 excited states of C5 and C8 are all dark states (f < 0.01), and only the lowest-energy absorption bands of C1 and C3 extend to visible-light region (at approximately 429 nm and 606 nm, respectively.). These two absorption exhibit LE and CT characters, respectively. Hence, solvent polarity exerts dramatically different influences on these two absorption bands. Compared to their positions in the gas phase, these absorption bands are blue-shifted about 20 nm and 220 nm in water, respectively.

Reactivity analysis identifies C7 as the most bifunctional isomer, its B–B bond serve as the electrophilic center (LEAE = −2.93 eV), and the surrounding framework as nucleophilic domains, positioning C7 as a promising candidate for catalysis and molecular recognition.

Overall, this work establishes a comprehensive structure–property map for B_6_C_6_N_6_ isomers, providing guidance for the rational design of BCN-based nanorings with tailored functionalities.

## Figures and Tables

**Table 1:** Thermodynamic parameters of eight B_6_C_6_N_6_ isomers at 298.15 K (E_Total_ and G are in a.u., ΔE and ΔG are in kcal/mol, C_V_, C_P_ and S are in cal/mol/K).

	E_Total_	ΔE	G	ΔG	C_V_	C_P_	S	Point Group
C1	−706.2951397	0.00	−706.337105	0.00	56.33	58.32	127.42	C_s_
C2	−706.2479177	29.63	−706.289049	30.16	56.19	58.18	125.79	C_3h_
C3	−706.1259060	106.19	−706.166932	106.79	58.45	60.44	126.12	C_2v_
C4	−706.0004269	184.93	−706.041307	185.62	59.13	61.12	125.96	C_2v_
C5	−705.9593956	210.68	−705.998947	212.20	59.70	61.69	123.17	D_3h_
C6	−705.9569187	212.23	−705.997647	213.01	59.46	61.45	125.67	C_2v_
C7	−705.9424878	221.29	−705.983294	222.02	59.61	61.59	125.98	C_2v_
C8	−705.9165912	237.54	−705.956410	238.89	60.12	62.10	124.19	D_3h_

Note: E_Total_ is Zero-point energy corrected total energy. All isomers are sorted by the relative Gibbs free energy (ΔG) at 298.15 K. The minimum-energy structure was set to zero for ΔE and ΔG.

**Table 2:** Hirshfeld charge separation (Δq = q(B) − q(N)); number of B–C, B–B, and N–N bonds; and relative energies (ΔE) for the eight B_6_C_6_N_6_ isomers.

Isomer	Δq (e)	B–C Bonds	B–B Bonds	N–N Bonds	μ/Debye
C1	0.592	1	0	0	2.65
C2	0.543	3	0	0	0
C3	0.498	2	0	1	9.92
C4	0.389	2	1	2	7.33
C5	0.325	0	3	0	0
C6	0.333	2	2	1	5.11
C7	0.333	4	1	2	4.81
C8	0.327	6	0	3	0

**Table 3:** Energies (in eV) of HOMO, LUMO, and energy gap of C1-C8.

	C1	C2	C3	C4	C5	C6	C7	C8
E_HOMO_	−8.07	−8.93	−6.92	−7.78	−8.25	−7.87	−8.16	−8.63
E_LUMO_	−1.17	−0.48	−2.52	−2.28	−0.87	−1.36	−1.63	−0.82
E_gap_	6.90	8.45	4.40	5.50	7.37	6.50	6.53	7.81

**Table 4:** Isovalues of ELF-π_in_, ELF-π_out_ and ELF-π isosurfaces of compounds C1–C8 (All ELF values are dimensionless).

	C1	C2	C3	C4	C5	C6	C7	C8
ELF-π_in_	0.310	0.368	0.185	0.165	0.338	0.195	0.215	0.225
ELF-π_out_	0.625	0.655	0.275	0.235	0.235	0.235	0.255	0.305
ELF-π	0.227	0.265	0.165	0.166	0.213	0.160	0.163	0.181

**Table 5:** Excited energy (in eV), excited wavelength (in nm), oscillator strength (*f*, dimensionless), and main molecular orbital transitions (with percentage contributions in parentheses) of selected excited states with obvious UV–Vis absorption characteristics.

	State	Energy/eV	λ/nm	*f*	Main MO Transitions
C1	S2	2.8934	428.51	0.01200	H→L (56.8%), H−1→L+1 (37.1%)
	S4	4.5539	272.26	0.99720	H−1→L+1 (51.0%), H→L (32.5%), H−2→L+2 (8.2%)
C2	S6	4.5229	274.13	0.06090	H−1→L+1 (16.1%), H→L (16.1%), H−4→L+1 (13.0%), H−5→L+2 (10.0%), and others
	S7	4.5229	274.13	0.06090	H→L+1 (16.1%), H−1→L (16.1%), H−4→L (13.0%), H−5→L+3 (10.0%), and others
C3	S2	2.0467	605.78	0.00950	H−1→L (50.1%), H→L+1 (48.9%)
	S4	2.2841	542.81	0.11350	H→L+1 (50.5%), H−1→L (49.4%)
C4	S6	3.3085	374.74	0.24640	H→L+1 (56.4%); H−1→L (42.6%)
C6	S12	4.3164	287.24	0.32840	H−1→L (51.9%); H→L+1 (39.5%); H−3→L+2 (6.1%)
C7	S10	4.0305	307.61	0.02390	H−3→L (31.8%); H−2→L+1 (30.2%); H→L+3 (22.9%); H−1→L+2 (8.5%)
	S11	4.3676	283.87	0.43670	H−1→L+1 (48.9%); H→L (48.1%)

Note: H and L denote HOMO and LUMO, respectively.

**Table 6:** Key electronic descriptors for selected excited states of C1–C8 at gas phase (HDI and EDI are dimensionless).

		Sr (a.u.)	D (Å)	t (Å)	H (Å)	HDI	EDI	E_coul_ (eV)	
C1	S2	0.66	0.90	−1.31	2.96	8.14	7.23	5.41	LE
	S4	0.73	1.29	−1.38	3.50	6.56	5.75	4.52	LE
C2	S6	0.75	0.35	−2.38	3.68	6.60	5.28	4.39	LE
	S7	0.75	0.35	−1.78	3.67	6.60	5.28	4.46	LE
C3	S2	0.31	5.02	3.71	2.78	9.61	5.68	2.71	CT
	S4	0.28	5.18	3.92	2.69	9.80	5.89	2.63	CT
C4	S6	0.36	4.70	3.29	3.16	7.71	5.88	2.81	CT
C6	S12	0.56	3.89	1.82	3.50	7.88	4.53	3.38	LE
C7	S10	0.75	1.55	−1.23	3.97	5.62	4.26	4.02	LE
	S11	0.52	4.26	2.30	3.34	6.58	5.72	3.25	CT

Note: Sr, D, t, H, HDI, EDI, and Ecoul denote Sr index (integral of Sr function), D index, H index, t index, hole delocalization index, electron delocalization index, and Coulomb attractive energy, respectively.

**Table 7:** Calculated excited-state parameters of C1 and C3 in gas phase and various solvents.

C1					
	State	Energy/eV	λ/nm	*f*	Main MO transitions
Gas phase	2	2.8934	428.51	0.01200	H→L (56.8%), H−1→L+1 (37.1%)
	4	4.5539	272.26	0.99720	H−1→L+1 (51.0%), H→L (32.5%), H−2→L+2 (8.2%)
*c*-C_6_H_6_	2	2.9258	423.76	0.0235	H→L (58.7%), H−1→L+1 (35.2%)
	4	4.3743	283.44	1.52650	H−1→L+1 (56.4%), H→L (33.3%)
CH_3_CN	2	3.0300	409.19	0.0235	H→L (58.6%), H−1→L+1 (35.2%)
	4	4.5088	274.98	1.4606	H−1→L+1 (55.3%), H→L (33.0%)
H_2_O	2	3.0422	407.55	0.0230	H→L (58.3%), H−1→L+1 (35.3%)
	4	4.5282	273.80	1.4518	H−1→L+1 (54.9%), H→L (33.0%), H−2→L+2 (5.1%)
**C3**					
	**State**	**Energy/eV**	**λ/nm**	***f***	**Main MO transitions**
Gas phase	2	2.0467	605.78	0.00950	H−1→L (50.1%), H→L+1 (48.9%)
	4	2.2841	542.81	0.11350	H→L+1 (50.5%), H−1→L (49.4%)
*c*-C_6_H_6_	2	2.5188	492.24	0.0393	H→L+1 (72.7%), H−1→L (26.1%)
	4	2.6794	462.73	0.1023	H−1→L (73.3%), H→L+1 (26.4%)
CH_3_CN	2	3.2548	380.93	0.0731	H→L+1 (91.9%), H−1→L (6.5%)
	5	3.4613	358.20	0.0473	H−1→L (92.7%), H→L+1 (6.8%)
H_2_O	3	3.2817	377.80	0.0737	H→L+1 (92.5%), H−1→L (6.0%)
	6	3.4886	355.40	0.0433	H−1→L (93.3%), H→L+1 (6.2%)

Note: State labels correspond to the order of excited states in each solvent environment. H and L denote HOMO and LUMO, respectively.

## Data Availability

The original contributions presented in this study are included in the article/Supplementary Materials. Further inquiries can be directed to the corresponding author.

## Supplementary Materials

The following supporting information can be downloaded at https://www.mdpi.com/article/10.3390/nano16150953/s1, Table S1: Optimized Cartesian coordinates (in Å) and the electronic energies of all studied molecules at the ωB97XD/def2-TZVP level. Table S2: B-centered bond angles of the eight B_6_C_6_N_6_ isomers. Table S3: N-centered bond angles of the eight B_6_C_6_N_6_ isomers. Table S4: Calculated Energies and Binding Energies of B_6_C_6_N_6_ Isomers at ωB97XD/def2-TZVP Level. Figure S1: Simulated IR spectra of compounds C1–C8 under gas phase. Table S5: Gibbs free energy changes at different temperatures (200–1000 K) of C1–C8. Table S6: Hirshfeld atomic charges (e) and ADCH atomic charges (e) of the eight B_6_C_6_N_6_ isomers. Figure S2: Isosurfaces of the HOMO and LUMO for compounds C1–C8. Figure S3: Total and partial density of states (TDOS and PDOS) plots of compounds C1. Figure S4: IRI-π isosurface maps of C1–C8. Figure S5: LOL (Localized Orbital Locator)-πin and LOL-πout isosurfaces of compounds C1–C8. Figure S6: Isosurface maps of ELF-π, ELF-π_in_ and ELF-π_out_ of B_6_C_6_N_6_ isomers. Table S7: Excited energy, excited wavelength, oscillator strength and main molecular orbital transitions of the first 20 excited states of C1–C8 at gas phase. Figure S7: The isosurface of hole and electron distribution, isosurface of C_ele_ and C_hole_ functions and isosurface of overlap of hole-electron (Sr) of various excitation. Figure S8: Simulated IR spectra of compound C1 and C3 in solvents. Table S8: Excited energy, excited wavelength, oscillator strength and main molecular orbital transitions of the first 20 excited states of C1 and C3 in solvents. Table S9: Selected excited-state descriptors for C1 and C3 in the gas phase, cyclohexane, acetonitrile, and water. Figure S9: Simulated UV-Vis absorption spectra of compounds C1 and C3 in solvents. Figure S10: Isosurfaces of the orbital-weighted dual descriptor for compounds C1–C8. Figure S11: Color-filled maps of the orbital-weighted dual descriptor isosurfaces.

## Abbreviations

The following abbreviations are used in this manuscript:

HOMO	The highest occupied molecular orbitals
LUMO	The lowest unoccupied molecular orbitals
FMO	Frontier molecular orbitals
ELF	Electron localization function
LOL	Localized orbital locator
OWDD	Orbital-weighted dual descriptor
HDI	Hole delocalization index
EDI	Electron delocalization index
Ecoul	Coulomb attractive energy
LEAE	Local atomic electron energy
