# Supplementary material for: Positional Isomers of B6C6N6 Nanorings: Stability, Reactivity, and Optical Properties from First Principles
Source: Nanomaterials (Basel). 2026 Aug 3;16(15):953. doi: 10.3390/nano16150953 (PMC13468401; doi:10.3390/nano16150953)
Supplement: Supplementary file 1 [file nanomaterials-16-00953-s001.zip › nanomaterials-4429458-supplementary.pdf]

Supplementary Materials

Positional Isomers of $B_6C_6N_6$ Nanorings: Stability, Reactivity, and Optical Properties from First-Principles

Xin Chen [1,2], Pei-pei Li [1,2,*] and Shu-sheng Gong [1,2,*]

1 School of materials and chemical engineering, West Anhui University, China 237012

2 Anhui Province Key Laboratory of Conservation and Utilization for Dabie Mountain Special Bio-Resources, West Anhui University, Lu'an, 237012, China

Table S1. Optimized Cartesian coordinates (in Å) and the electronic energies of all studied molecules at the ωB97XD/def2-TZVP level

Table S2. B-centered bond angles of the eight $B_6C_6N_6$ isomers

Table S3. N-centered bond angles of the eight $B_6C_6N_6$ isomers

Table S4. Calculated Energies and Binding Energies of $B_6C_6N_6$ Isomers at ωB97XD/def2-TZVP Level

Figure S1. Simulated IR spectra of compounds C1-C8 under gas phase.

Table S5. Gibbs free energy changes at different temperatures (200-1000 K) of C1-C8

Table S6. Hirshfeld atomic charges (e) and ADCH atomic charges (e) of the eight $B_6C_6N_6$ isomers.

Figure S2. Isosurfaces of the HOMO and LUMO for compounds C1–C8

Figure S3. Total and partial density of states (TDOS and PDOS) plots of compounds C1.

Figure S4. IRI-π isosurface maps of C1-C8.

Figure S5. LOL (Localized Orbital Locator)-π_{in} and LOL-π_{out} isosurfaces of compounds C1-C8.

Figure S6. Isosurface maps of ELF-π, ELF-π_{in} and ELF-π_{out} of $B_6C_6N_6$ isomers.

Table S7. Excited energy, excited wavelength, oscillator strength and main molecular orbital transitions of the first 20 excited states of C1-C8 at gas phase.

Figure S7. The isosurface of hole and electron distribution, isosurface of C_{ele} and C_{hole} functions and isosurface of overlap of hole-electron (Sr) of various excitation.

Figure S8. Simulated IR spectra of compound C1 and C3 in solvents.

Table S8. Excited energy, excited wavelength, oscillator strength and main molecular orbital transitions of the first 20 excited states of C1 and C3 in solvents.

Table S9. Selected excited-state descriptors for C1 and C3 in the gas phase, cyclohexane, acetonitrile, and water.

Figure S9. Simulated UV-Vis absorption spectra of compounds C1 and C3 in solvents.

Figure S10. Isosurfaces of the orbital-weighted dual descriptor for compounds C1-C8.

Figure S11. Color-filled maps of the orbital-weighted dual descriptor isosurfaces.

Table S1 Optimized Cartesian coordinates (in Å) and the electronic energies of all studied molecules at the ωB97XD/def2-TZVP level.

**C1** (charge = 0, spin multiplicity = 1) E = -706.277511 a.u.			
C	3.39782400	1.91166900	0.00000000
C	2.38735000	2.61789700	0.00000000
B	3.82847700	0.54990700	0.00000000
B	-1.19355000	-3.17180700	0.00000000
N	4.07738700	-0.72441800	0.00000000
N	-2.43192600	-2.74584800	0.00000000
C	-2.38727900	2.67049000	0.00000000
C	-1.32990600	3.30462400	0.00000000
C	0.00000000	3.34841200	0.00000000
C	1.22839200	3.27729000	0.00000000
B	3.33870100	-1.82512100	0.00000000
B	1.31256200	-3.24599400	0.00000000
B	-3.18282000	-1.66927800	0.00000000
B	-3.70742800	0.71828400	0.00000000
N	2.58082000	-2.88710000	0.00000000
N	0.05200800	-3.58919000	0.00000000
N	-3.91471700	-0.57780500	0.00000000
N	-3.47185800	2.01546900	0.00000000

**C2** (charge = 0, spin multiplicity = 1) E = -706.230227 a.u.			
C	1.23520700	3.51581200	0.00000000
C	0.00000000	3.58153900	0.00000000
C	-3.66238600	-0.68818500	0.00000000
C	-3.10170400	-1.79077000	0.00000000
C	3.10170400	-1.79077000	0.00000000
C	2.42717900	-2.82762600	0.00000000
B	1.13908800	-3.40768400	0.00000000
B	-1.32063700	-3.39338700	0.00000000
B	-3.52068400	0.71736300	0.00000000
B	-2.27844000	2.84039900	0.00000000
B	2.38159700	2.69032100	0.00000000
B	3.59907800	0.55298800	0.00000000
N	-0.08975000	-3.85104100	0.00000000
N	-2.54770400	-2.92312100	0.00000000
N	-3.29022400	2.00324600	0.00000000
N	-1.25764400	3.66793700	0.00000000
N	3.37997400	1.84779400	0.00000000
N	3.80534900	-0.74481600	0.00000000

**C3** (charge = 0, spin multiplicity = 1) E = -706.107952 a.u.			
C	0.00000000	2.97786000	2.41706600
C	0.00000000	1.91008900	2.99221000
B	0.00000000	3.79848100	1.19919900
N	0.00000000	4.27780300	0.02749400
C	0.00000000	-2.97786000	2.41706600
C	0.00000000	-1.91008900	2.99221000
C	0.00000000	-0.60531400	3.36378100
C	0.00000000	0.60531400	3.36378100
B	0.00000000	3.74669600	-1.23460000
B	0.00000000	1.84714100	-2.82132900
N	0.00000000	3.14552200	-2.35394400
N	0.00000000	0.64546900	-3.15279200

B	0.00000000	-3.79848100	1.19919900
B	0.00000000	-3.74669600	-1.23460000
B	0.00000000	-1.84714100	-2.82132900
N	0.00000000	-4.27780300	0.02749400
N	0.00000000	-3.14552200	-2.35394400
N	0.00000000	-0.64546900	-3.15279200

**C4** (charge = 0, spin multiplicity = 1) E = -705.982401 a.u.			
C	0.00000000	2.97684900	2.45967100
C	0.00000000	1.91115900	3.03924800
B	0.00000000	3.78837300	1.23344600
N	0.00000000	4.23448900	0.05083900
C	0.00000000	-2.97684900	2.45967100
C	0.00000000	-1.91115900	3.03924800
C	0.00000000	-0.60546800	3.41027600
C	0.00000000	0.60546800	3.41027600
B	0.00000000	3.68233100	-1.20953500
N	0.00000000	3.06570100	-2.30249300
B	0.00000000	-3.78837300	1.23344600
B	0.00000000	-3.68233100	-1.20953500
N	0.00000000	-4.23448900	0.05083900
N	0.00000000	-3.06570100	-2.30249300
B	0.00000000	-0.80567800	-3.40445100
B	0.00000000	0.80567800	-3.40445100
N	0.00000000	-1.96701400	-2.97012800
N	0.00000000	1.96701400	-2.97012800

**C5** (charge = 0, spin multiplicity = 1) E = -705.941368 a.u.			
C	0.60345800	3.60350300	0.00000000
C	-0.60345800	3.60350300	0.00000000
C	-3.42245400	-1.27914200	0.00000000
C	-2.81899600	-2.32436100	0.00000000
C	3.42245400	-1.27914200	0.00000000
C	2.81899600	-2.32436100	0.00000000
N	-3.81530500	-0.03786300	0.00000000
N	-1.87486300	3.32308300	0.00000000
N	1.87486300	3.32308300	0.00000000
N	3.81530500	-0.03786300	0.00000000
N	1.94044300	-3.28522000	0.00000000
N	-1.94044300	-3.28522000	0.00000000
B	0.80593700	-3.81020400	0.00000000
B	-0.80593700	-3.81020400	0.00000000
B	-3.70270200	1.20714000	0.00000000
B	-2.89676500	2.60306400	0.00000000
B	2.89676500	2.60306400	0.00000000
B	3.70270200	1.20714000	0.00000000

**C6** (charge = 0, spin multiplicity = 1) E = -705.938880 a.u.			
C	3.37044900	-1.82004500	0.00000000
C	3.70612000	-0.65312600	0.00000000
C	0.00000000	3.60509100	0.00000000
C	-1.18992300	3.40349000	0.00000000
C	-2.58080300	-2.82911800	0.00000000
C	-3.28280100	-1.83837200	0.00000000
N	1.29858800	3.54485300	0.00000000
N	3.88806300	0.62904000	0.00000000

N	-3.87794200	-0.68820400	0.00000000
N	-2.39616000	2.91872100	0.00000000
B	-3.89965100	0.56541400	0.00000000
B	-3.30793700	2.06569600	0.00000000
B	2.44066800	3.04025700	0.00000000
B	3.49456100	1.81947100	0.00000000
N	1.23834700	-3.46695600	0.00000000
N	-0.02487200	-3.68101800	0.00000000
B	2.31620500	-2.84363500	0.00000000
B	-1.24792900	-3.44771400	0.00000000

**C7** (charge = 0, spin multiplicity = 1) E = -705.924381 a.u.			
C	0.00000000	0.60662200	-3.79480800
C	0.00000000	-0.60662200	-3.79480800
C	0.00000000	-3.57561400	1.40117400
C	0.00000000	-2.86294700	2.38398300
C	0.00000000	3.57561400	1.40117400
C	0.00000000	2.86294700	2.38398300
B	0.00000000	-3.72704400	-0.05885300
B	0.00000000	1.96230300	-3.20833500
N	0.00000000	-3.55542300	-1.29419300
N	0.00000000	2.89273700	-2.38776700
N	0.00000000	-2.89273700	-2.38776700
N	0.00000000	3.55542300	-1.29419300
B	0.00000000	-1.96230300	-3.20833500
B	0.00000000	3.72704400	-0.05885300
B	0.00000000	-0.80784700	3.78890200
B	0.00000000	0.80784700	3.78890200
N	0.00000000	-1.96731300	3.31758100
N	0.00000000	1.96731300	3.31758100

**C8** (charge = 0, spin multiplicity = 1) E = -705.898349 a.u.			
C	0.60640000	-3.81047000	0.00000000
C	-0.60640000	-3.81047000	0.00000000
C	-3.60316400	1.38007700	0.00000000
C	-2.99676400	2.43039300	0.00000000
C	3.60316400	1.38007700	0.00000000
C	2.99676400	2.43039300	0.00000000
B	1.81055900	3.30982500	0.00000000
B	-3.77167200	-0.08692300	0.00000000
B	1.96111300	-3.22290200	0.00000000
N	0.63772500	3.71824100	0.00000000
N	-3.53895400	-1.30683400	0.00000000
N	2.90122900	-2.41140700	0.00000000
N	-2.90122900	-2.41140700	0.00000000
N	3.53895400	-1.30683400	0.00000000
N	-0.63772500	3.71824100	0.00000000
B	-1.81055900	3.30982500	0.00000000
B	-1.96111300	-3.22290200	0.00000000
B	3.77167200	-0.08692300	0.00000000

Note: The SCF (Self-Consistent Field) convergence criteria for RMS (root-mean-square) density matrix, maximum density matrix and energy are 1.00×10^{-8}, 1.00×10^{-6}, and 1.00×10^{-6} Hartree, respectively. The geometry optimization convergence tolerances for maximum force, RMS force, maximum displacement and RMS displacement are 4.50×10^{-4} Hartree/Bohr, 3.00×10^{-4} Hartree/Bohr, 1.80×10^{-3} Bohr and 1.20×10^{-3} Bohr, respectively. All structures are true minima, as no imaginary frequencies were observed.

Table S2 B-centered bond angles of the eight $B_6C_6N_6$ isomers

Isome	Bond angle type	Values (°)	Number
C1	N–B–N	178.79, 178.95, 179.54, 179.43, 178.35	5
	C–B–N	173.50	1
C2	N–B–N	179.43	3
	C–B–N	175.60	3
C3	N–B–N	174.58, 175.62	2 each
	C–B–N	168.28	2
C4	N–B–N	174.23	2
	C–B–N	167.17	2
	N–B–B	159.49	2
C5	N–B–B	155.17	6
C6	N–B–B	154.62, 157.49	2 each
	C–B–N	165.90	2
C7	N–B–B	157.87	2
	C–B–N	161.98, 166.17	2 each
C8	C–B–N	162.64	6

Table S3 N-centered bond angles of the eight $B_6C_6N_6$ isomers

Isome	Bond angle type	Values (°)	Number
C1	C-N–B	131.42	1
	B–N–B	137.07, 143.88, 146.24, 141.31, 135.08	5
C2	B–N–B	139.77	3
	C-N–B	137.04	3
C3	B–N–B	134.93, 138.04	2 each
	B–N–N	164.58	2
C4	B–N–B	135.67	2
	B–N–N	150.72, 169.22	2 each
C5	C-N–B	157.27	6
C6	C-N–B	158.82, 153.63	2 each
	B–N–N	159.58	2
C7	C-N–B	155.93	2
	B–N–N	162.62, 156.69	2 each
C8	B–N–N	160.80	6

Table S4. Calculated Energies and Binding Energies of $B_6C_6N_6$ Isomers at ωB97XD/def2-TZVP Level

Isomer	E (a.u.)	ZPE (a.u.)	E_{Total} (a.u.)	ΔE	ΔE_{bind} (kcal/mol)	Average ΔE_{bind} (eV/atom)
C1	-706.3771627	0.082023	-706.2951397	0.00	-2393.61	-5.77
C2	-706.3298847	0.081967	-706.2479177	29.63	-2363.97	-5.70
C3	-706.2068730	0.080967	-706.1259060	106.19	-2287.41	-5.51
C4	-706.0810009	0.080574	-706.0004269	184.93	-2208.67	-5.32
C5	-706.0400216	0.080626	-705.9593956	210.68	-2182.92	-5.26
C6	-706.0375687	0.080650	-705.9569187	212.23	-2181.37	-5.26
C7	-706.0229898	0.080502	-705.9424878	221.29	-2172.31	-5.23
C8	-705.9967982	0.080207	-705.9165912	237.54	-2156.06	-5.19

Note: ZPE values are scaled by a factor of 0.9596 at ωB97X-D/def2-TZVP level. E_{Total} = E + ZPE. The definition of ΔE_{bind} and average ΔE_{bind} are shown in Computational Details of main text.

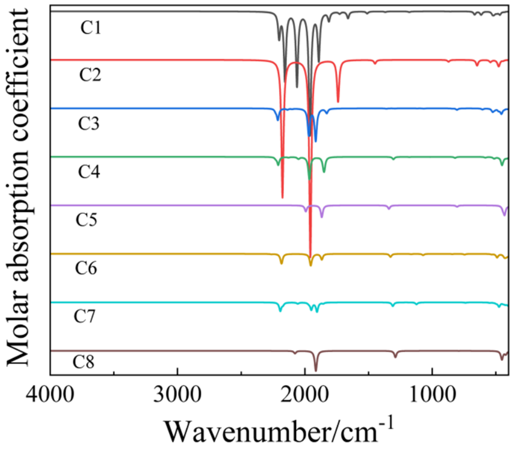

Figure S1. Simulated IR spectra of compounds C1-C8 under gas phase calculated at ωB97X-D/def2-TZVP level.

Table S5 Gibbs free energy changes at different temperatures (200-1000 K) of C1-C8

	C1	C2	C3	C4	C5	C6	C7	C8
200 K	11.04	41.12	117.73	195.00	212.13	213.18	222.27	240.02
300 K	-0.23	30.16	106.79	185.62	212.20	213.01	222.02	238.89
400 K	-12.99	17.32	94.51	175.30	211.46	211.76	219.90	237.57
500 K	-27.54	2.90	80.73	163.51	210.02	209.54	217.02	235.96
600 K	-43.51	-13.04	65.30	149.98	207.86	206.28	213.26	234.03
700 K	-60.73	-30.23	48.01	134.53	204.95	201.91	208.53	231.75
800 K	-79.01	-48.51	28.71	116.96	201.22	196.28	202.65	229.08
900 K	-98.22	-67.72	7.24	97.03	196.59	189.28	195.45	225.97
1000 K	-118.22	-87.72	-17.56	74.49	190.98	180.76	186.78	222.37

Note: $\Delta G_i(T)$ is defined as $G_i(T)$-G_{C1}(298.15 K), where G_{C1}(298.15 K)= -706.3371054 a.u., it is the absolute Gibbs free energy of isomer C1 at 298.15 K, serving as a fixed reference point for all temperatures. The temperature dependence of ΔG reflects the cumulative differences in entropy and heat capacity among the isomers, which are amplified at high temperatures under this fixed-reference definition.

Table S6(a) Hirshfeld atomic charges (e) of the eight $B_6C_6N_6$ isomers. Atomic labels correspond to the connectivity sequences given in **Figure 1**.

Atoms of C1	charge	Atoms of C2	charge	Atoms of C3	charge	Atoms of C4	charge
C1	-0.17	C1	-0.22	C1	-0.06	C1	-0.07
C2	0.06	C2	0.12	C2	0.05	C2	0.04
B3	0.29	C3	-0.22	B3	0.30	B3	0.28
B4	0.31	C4	0.12	N4	-0.26	N4	-0.26
N5	-0.28	C5	0.12	C5	-0.06	C5	-0.07
N6	-0.30	C6	-0.22	C6	0.05	C6	0.04
C7	0.10	B7	0.27	C7	0.03	C7	0.02
C8	-0.13	B8	0.32	C8	0.03	C8	0.02
C9	0.05	B9	0.27	B9	0.28	B9	0.23
C10	-0.09	B10	0.32	B10	0.17	N10	-0.18
B11	0.32	B11	0.27	N11	-0.30	B11	0.28
B12	0.31	B12	0.32	N12	-0.20	B12	0.23
B13	0.31	N13	-0.28	B13	0.30	N13	-0.26
B14	0.31	N14	-0.20	B14	0.28	N14	-0.18
N15	-0.30	N15	-0.28	B15	0.17	B15	0.07
N16	-0.30	N16	-0.20	N16	-0.26	B16	0.07
N17	-0.30	N17	-0.28	N17	-0.30	N17	-0.14
N18	-0.21	N18	-0.20	N18	-0.20	N18	-0.14
**Atoms of C5**	**charge**	**Atoms of C6**	**charge**	**Atoms of C7**	**charge**	**Atoms of C8**	**charge**
C1	-0.001	C1	-0.138	C1	-0.071	C1	-0.064
C2	-0.001	C2	0.057	C2	-0.071	C2	-0.064
C3	-0.001	C3	0.006	C3	-0.13	C3	-0.064
C4	-0.001	C4	0.006	C4	0.064	C4	-0.063
C5	-0.001	C5	-0.138	C5	-0.13	C5	-0.064
C6	-0.001	C6	0.057	C6	0.064	C6	-0.063
N7	-0.162	N7	-0.153	B7	0.186	B7	0.195
N8	-0.162	N8	-0.157	B8	0.177	B8	0.195
N9	-0.162	N9	-0.157	N9	-0.149	B9	0.195
N10	-0.162	N10	-0.153	N10	-0.135	N10	-0.132
N11	-0.162	B11	0.199	N11	-0.135	N11	-0.132
N12	-0.162	B12	0.171	N12	-0.149	N12	-0.132
B13	0.163	B13	0.171	B13	0.177	N13	-0.132
B14	0.163	B14	0.199	B14	0.186	N14	-0.132
B15	0.163	N15	-0.152	B15	0.206	N15	-0.132
B16	0.163	N16	-0.152	B16	0.206	B16	0.195
B17	0.163	B17	0.167	N17	-0.149	B17	0.195
B18	0.163	B18	0.167	N18	-0.149	B18	0.195

Table S6(b) Atomic dipole corrected Hirshfeld atomic charge (ADCH) (e) of the eight $B_6C_6N_6$ isomers. Atomic labels correspond to the connectivity sequences given in **Figure 1**.

Atoms of C1	charge	Atoms of C2	charge	Atoms of C3	charge	Atoms of C4	charge
C1	-0.16	C1	-0.25	C1	-0.08	C1	-0.08
C2	0.04	C2	0.09	C2	0.05	C2	0.04
B3	0.28	C3	-0.25	B3	0.31	B3	0.30
B4	0.27	C4	0.09	N4	-0.22	N4	-0.22
N5	-0.22	C5	0.09	C5	-0.08	C5	-0.08
N6	-0.27	C6	-0.25	C6	0.05	C6	0.04
C7	0.07	B7	0.27	C7	0.04	C7	0.03
C8	-0.13	B8	0.28	C8	0.04	C8	0.03
C9	0.04	B9	0.27	B9	0.23	B9	0.24
C10	-0.06	B10	0.28	B10	0.19	N10	-0.25
B11	0.27	B11	0.27	N11	-0.28	B11	0.30
B12	0.28	B12	0.28	N12	-0.24	B12	0.24
B13	0.27	N13	-0.23	B13	0.31	N13	-0.22
B14	0.29	N14	-0.17	B14	0.23	N14	-0.25
N15	-0.26	N15	-0.23	B15	0.19	B15	0.14
N16	-0.26	N16	-0.17	N16	-0.22	B16	0.14
N17	-0.26	N17	-0.23	N17	-0.28	N17	-0.19
N18	-0.19	N18	-0.17	N18	-0.24	N18	-0.19
**Atoms of C5**	**charge**	**Atoms of C6**	**charge**	**Atoms of C7**	**charge**	**Atoms of C8**	**charge**
C1	-0.02	C1	-0.20	C1	-0.13	C1	-0.12
C2	-0.02	C2	0.03	C2	-0.13	C2	-0.12
C3	-0.02	C3	-0.02	C3	-0.19	C3	-0.12
C4	-0.02	C4	-0.02	C4	0.03	C4	-0.12
C5	-0.02	C5	-0.20	C5	-0.19	C5	-0.12
C6	-0.02	C6	0.03	C6	0.03	C6	-0.12
N7	-0.20	N7	-0.19	B7	0.35	B7	0.37
N8	-0.20	N8	-0.18	B8	0.34	B8	0.37
N9	-0.20	N9	-0.18	N9	-0.25	B9	0.37
N10	-0.20	N10	-0.19	N10	-0.24	N10	-0.24
N11	-0.20	B11	0.26	N11	-0.24	N11	-0.24
N12	-0.20	B12	0.23	N12	-0.25	N12	-0.24
B13	0.22	B13	0.23	B13	0.34	N13	-0.24
B14	0.22	B14	0.26	B14	0.35	N14	-0.24
B15	0.22	N15	-0.25	B15	0.26	N15	-0.24
B16	0.22	N16	-0.25	B16	0.26	B16	0.37
B17	0.22	B17	0.33	N17	-0.17	B17	0.37
B18	0.22	B18	0.33	N18	-0.17	B18	0.37

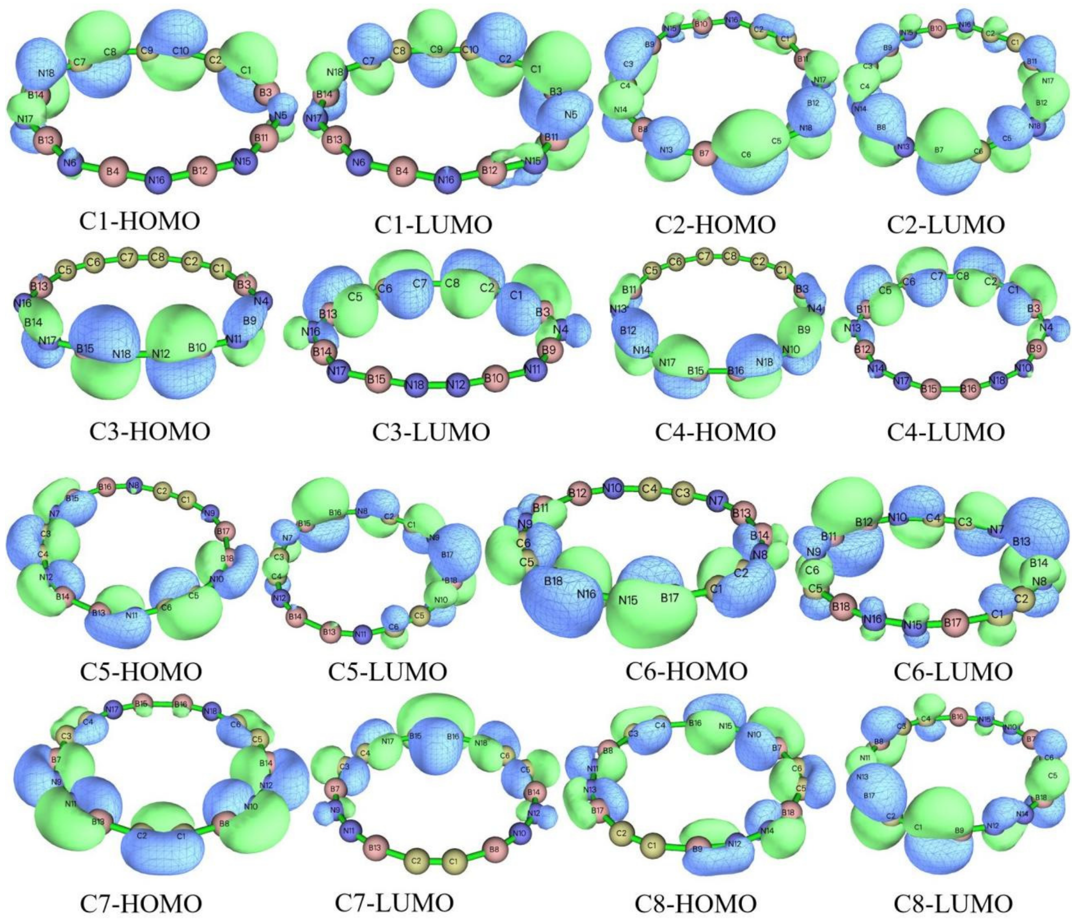

Figure S2. Isosurfaces of the HOMO and LUMO for compounds C1–C8 (isovalue = 0.05 a.u.).

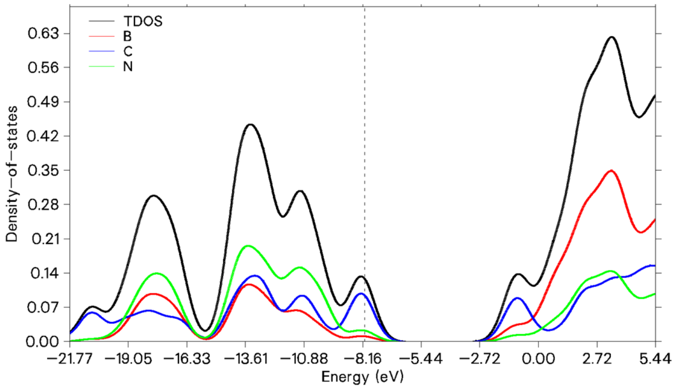

Figure S3 (a). Total and partial density of states (TDOS and PDOS) plots of compounds C1. PDOS analyses were performed using the Hirshfeld method, with a full width at half-maximum (FWHM) of 0.05 a.u. The TDOS is shown in black, while the PDOS for B, C, and N atoms are displayed in red, blue, and green, respectively.

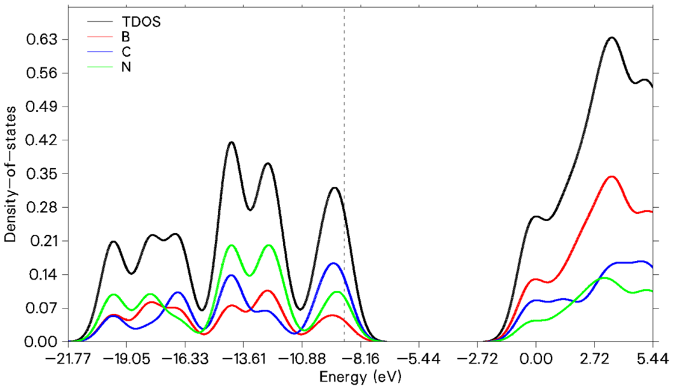

Figure S3 (b). Total and partial density of states (TDOS and PDOS) plots of compounds C2. PDOS analyses were performed using the Hirshfeld method, with a full width at half-maximum (FWHM) of 0.05 a.u. The TDOS is shown in black, while the PDOS for B, C, and N atoms are displayed in red, blue, and green, respectively.

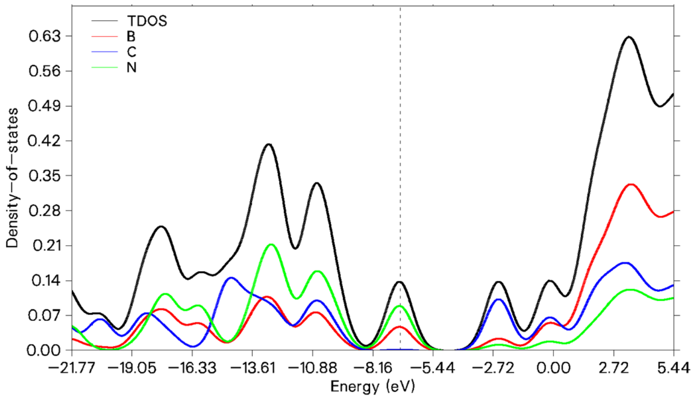

Figure S3 (c). Total and partial density of states (TDOS and PDOS) plots of compounds C3. PDOS analyses were performed using the Hirshfeld method, with a full width at half-maximum (FWHM) of 0.05 a.u. The TDOS is shown in black, while the PDOS for B, C, and N atoms are displayed in red, blue, and green, respectively.

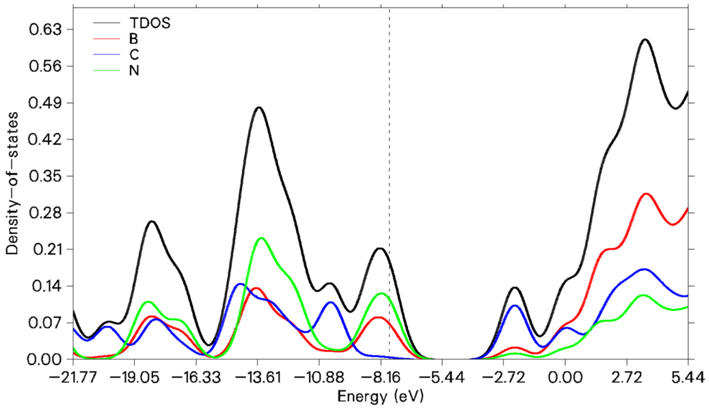

Figure S3 (d). Total and partial density of states (TDOS and PDOS) plots of compounds C4. PDOS analyses were performed using the Hirshfeld method, with a full width at half-maximum (FWHM) of 0.05 a.u. The TDOS is shown in black, while the PDOS for B, C, and N atoms are displayed in red, blue, and green, respectively.

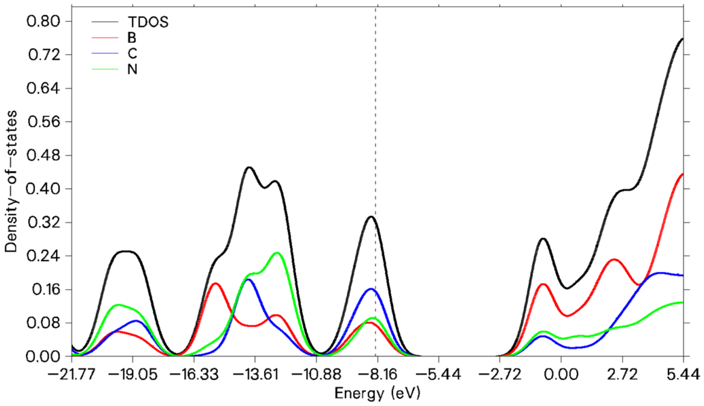

Figure S3 (e). Total and partial density of states (TDOS and PDOS) plots of compounds C5. PDOS analyses were performed using the Hirshfeld method, with a full width at half-maximum (FWHM) of 0.05 a.u. The TDOS is shown in black, while the PDOS for B, C, and N atoms are displayed in red, blue, and green, respectively.

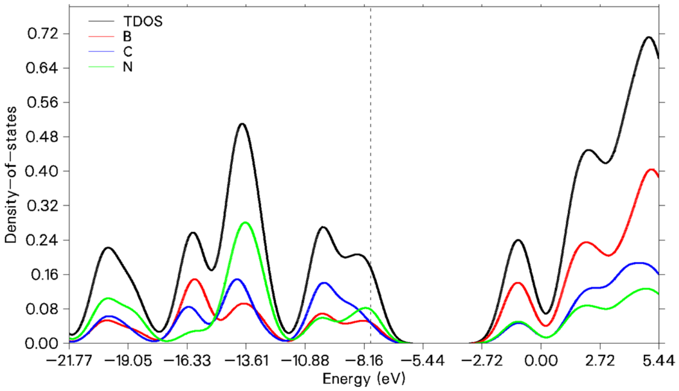

Figure S3 (f). Total and partial density of states (TDOS and PDOS) plots of compounds C6. PDOS analyses were performed using the Hirshfeld method, with a full width at half-maximum (FWHM) of 0.05 a.u. The TDOS is shown in black, while the PDOS for B, C, and N atoms are displayed in red, blue, and green, respectively.

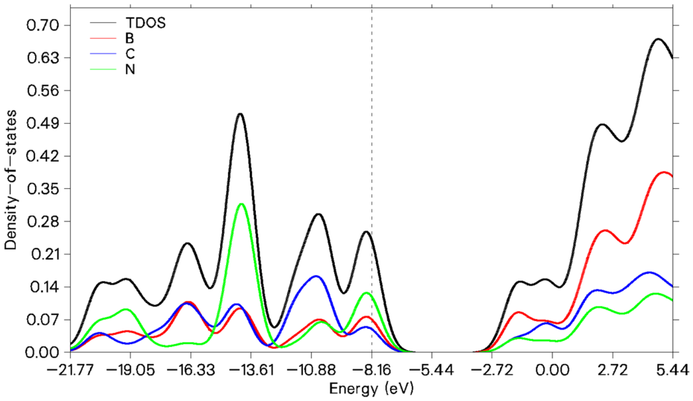

Figure S3 (g). Total and partial density of states (TDOS and PDOS) plots of compounds C7. PDOS analyses were performed using the Hirshfeld method, with a full width at half-maximum (FWHM) of 0.05 a.u. The TDOS is shown in black, while the PDOS for B, C, and N atoms are displayed in red, blue, and green, respectively.

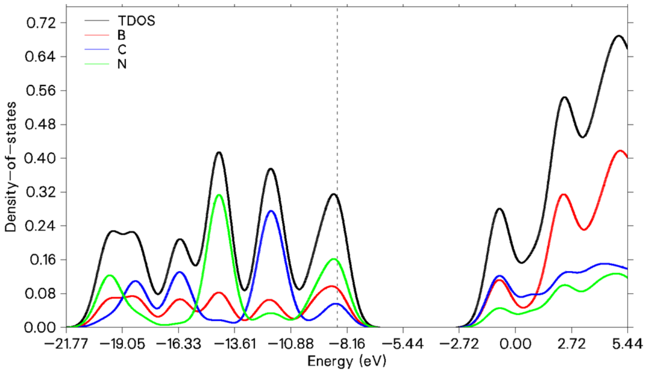

Figure S3 (h). Total and partial density of states (TDOS and PDOS) plots of compounds C8. PDOS analyses were performed using the Hirshfeld method, with a full width at half-maximum (FWHM) of 0.05 a.u. The TDOS is shown in black, while the PDOS for B, C, and N atoms are displayed in red, blue, and green, respectively.

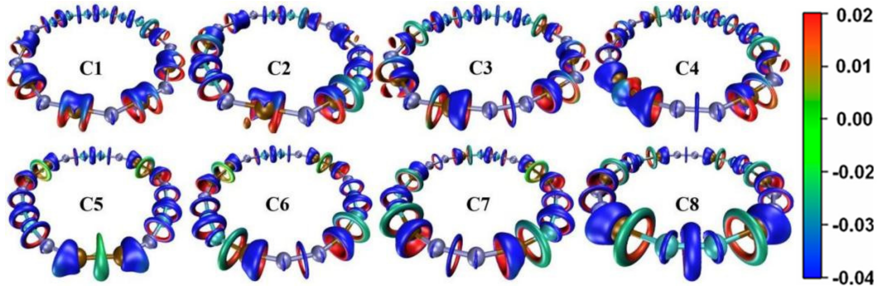

Figure S4. IRI-π isosurface maps of C1-C8 (colored by sign(λ2)ρ, isovalue = 1.0 a.u.). B, C and N atoms are in red, gray and blue, respectively. The color bar scale is in a.u..

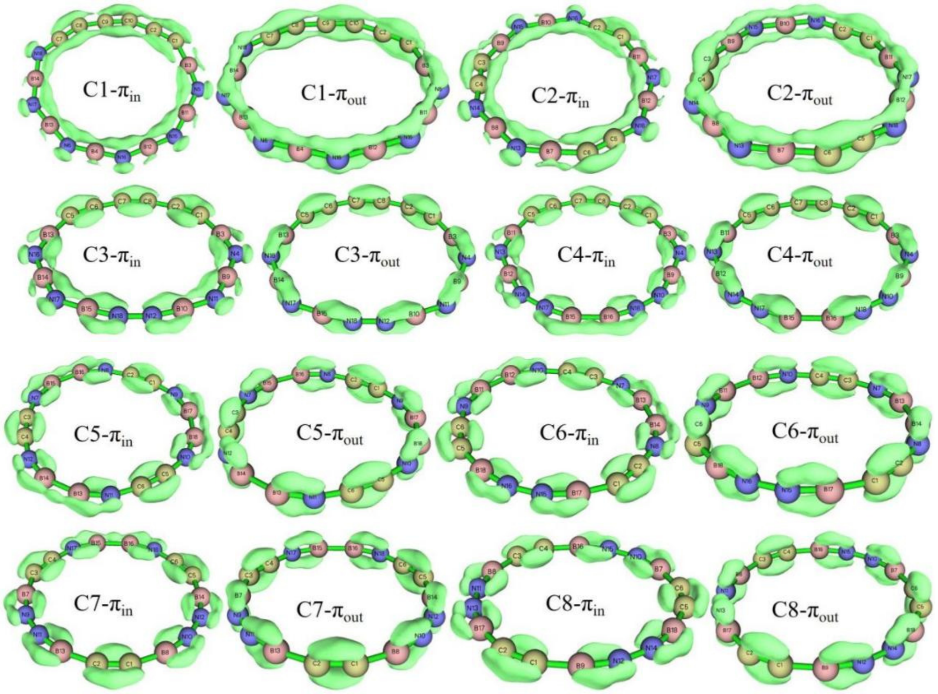

Figure S5. LOL (Localized Orbital Locator)-π_{in} and LOL-π_{out} isosurfaces of compounds C1-C8 (isovalue = 0.5 a.u.).

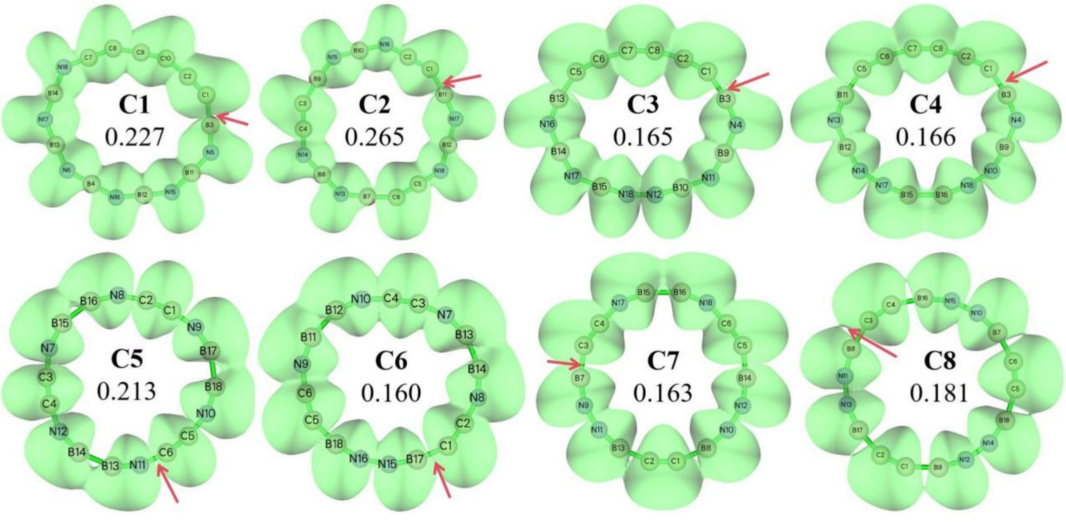

Figure S6(a) Isosurface maps of ELF-π of $B_6C_6N_6$ isomers (**C1-C8**).The values labeled on the graphs are bifurcation values.

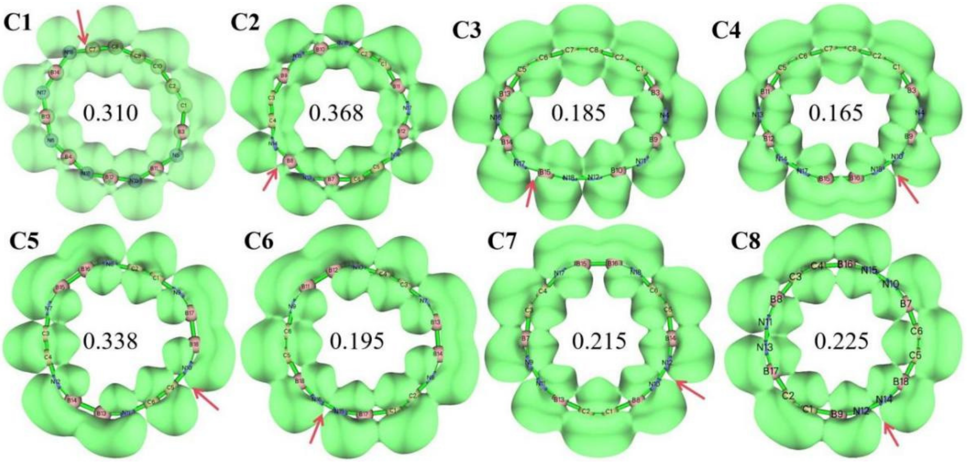

Figure S6(b) Isosurface maps of ELF-π_{in} of $B_6C_6N_6$ isomers (**C1-C8**).The values labeled on the graphs are bifurcation values.

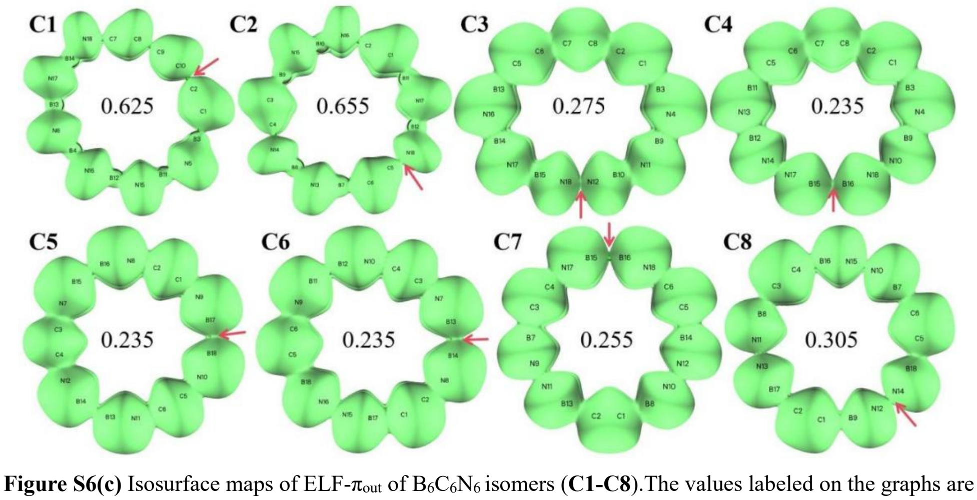

Figure S6(c) Isosurface maps of ELF-π_{out} of $B_6C_6N_6$ isomers (**C1-C8**).The values labeled on the graphs are bifurcation values.

Table S7 (a). Excited energy (in eV), excited wavelength (in nm), oscillator strength (*f*) and main molecular orbital transitions of 20 excited states of **C1** at gas phase

State	Energy/eV	λ/nm	f	Mayor MO transitions
1	2.7906	444.29	0.00000	H→L+1 (79.0%), H-1→L (14.7%)
**2**	**2.8934**	**428.51**	**0.01200**	**H→L (56.8%), H-1→L+1 (37.1%)**
3	2.9298	423.18	0.00050	H-1→L (78.8%), H→L+1 (15.0%)
**4**	**4.5539**	**272.26**	**0.99720**	**H-1→L+1 (51.0%), H→L (32.5%), H-2→L+2 (8.2%)**
5	4.8232	257.06	0.00000	H-5→L (16.7%), H-4→L+1 (16.0%), H-3→L (13.4%), H-2→L+1 (12.5%), H-1→L+2 (11.9%), H-1→L+8 (7.1%), H→L+6 (7.0%)
6	4.9490	250.52	0.05500	H→L+2 (18.6%), H-2→L (16.1%), H-4→L (14.7%), H-5→L+1 (13.1%), H-3→L+1 (9.9%), H→L+8 (7.0%), H-1→L+6 (6.2%)
7	4.9657	249.68	0.00000	H-4→L+1 (16.9%), H-2→L+1 (14.5%), H-5→L (13.7%), H-1→L+2 (12.7%), H-3→L (12.4%), H→L+6 (7.5%), H-1→L+8 (6.2%), H→L+3 (5.3%)
8	5.5947	221.61	0.05000	H→L+2 (51.4%), H-2→L (22.8%)
9	5.7679	214.96	0.00020	H→L+4 (34.3%), H→L+3 (29.5%), H→L+10 (9.7%), H→L+5 (9.0%)
10	5.8186	213.08	0.00030	H-1→L+2 (45.1%), H-3→L (16.5%), H-5→L (8.7%), H→L+4 (5.8%)
11	5.8877	210.58	0.00330	H→L+4 (21.7%), H→L+3 (18.7%), H-2→L+1 (14.6%), H→L+7 (8.3%), H-1→L+2 (6.5%), H-4→L+1 (6.0%)
12	6.0825	203.84	0.00000	H-3→L+2 (36.2%), H-7→L (18.4%), H-5→L (15.0%)
13	6.1399	201.93	0.36440	H-2→L+2 (20.9%), H-4→L (13.0%), H-2→L (8.7%), H-6→L (8.7%), H-5→L+1 (7.2%), H-3→L+3 (7.0%), H→L+2 (6.8%)
14	6.2055	199.80	0.00080	H→L+6 (30.9%), H-2→L+3 (17.4%), H→L+7 (9.1%), H-2→L+1 (5.4%)
15	6.2246	199.18	0.02050	H-1→L+4 (54.7%), H-1→L+3 (14.1%), H-1→L+10 (12.0%)
16	6.3293	195.89	0.35840	H-2→L (24.9%), H-2→L+2 (17.3%), H-1→L+6 (6.8%), H-3→L+3 (6.2%), H→L+8 (5.2%)
17	6.3483	195.30	0.00010	H-2→L+1 (14.2%), H-6→L+1 (12.2%), H-4→L+1 (9.1%), H-2→L+3 (7.8%), H-8→L+1 (5.6%)
18	6.4484	192.27	0.00010	H-3→L+2 (22.6%), H-3→L (22.2%), H-5→L (11.4%), H-7→L+2 (11.4%), H-3→L+9 (5.5%), H-9→L (5.3%)
19	6.5356	189.71	0.00000	H-2→L+3 (17.2%), H-4→L+3 (9.6%), H-11→L (7.4%), H-2→L+6 (7.3%), H-5→L+2 (6.7%), H-10→L+1 (6.0%)
20	6.5480	189.35	0.00070	H→L+5 (53.2%), H-2→L+4 (8.4%), H→L+4 (6.0%), H→L+6 (5.6%), H→L+12 (5.1%)

Table S7 (b). Excited energy (in eV), excited wavelength (in nm), oscillator strength (*f*) and main molecular orbital transitions of 20 excited states of **C2** at gas phase

State	Energy/eV	λ/nm	*f*	Mayor MO transitions
1	4.1328	300.00	0.00000	H-1→L+1 (40.8%), H→L (40.8%)
2	4.2733	290.14	0.00030	H-3→L+1 (29.1%), H-2→L (29.1%), H-2→L+1 (10.4%), H-3→L

				(10.4%), H-5→L+6 (7.7%)
3	4.3700	283.72	0.00040	H→L+3 (27.1%), H-1→L+2 (27.1%), H→L+2 (11.1%), H-1→L+3 (11.1%), H-4→L+4 (5.1%)
4	4.4470	278.80	0.00000	H-5→L+1 (12.8%), H-3→L+1 (10.4%), H-2→L (10.4%), H-3→L (10.0%), H-2→L+1 (10.0%), H-5→L (9.0%), H-2→L+6 (8.7%)
5	4.4470	278.80	0.00000	H-5→L (12.8%), H-2→L+1 (10.4%), H-3→L (10.4%), H-2→L (10.0%), H-3→L+1 (10.0%), H-5→L+1 (9.0%), H-3→L+6 (8.7%)
**6**	**4.5229**	**274.13**	**0.06090**	**H-1→L+1 (16.1%), H→L (16.1%), H-4→L+1 (13.0%), H-5→L+2 (10.0%), H-2→L+3 (5.6%), H-3→L+2 (5.6%), H-1→L+6 (5.6%)**
**7**	**4.5229**	**274.13**	**0.06090**	**H→L+1 (16.1%), H-1→L (16.1%), H-4→L (13.0%), H-5→L+3 (10.0%), H-2→L+2 (5.6%), H-3→L+3 (5.6%), H→L+6 (5.6%)**
8	4.5599	271.90	0.00000	H→L+2 (15.2%), H-1→L+3 (15.2%), H-4→L+3 (14.7%), H-1→L+4 (5.7%)
9	4.5599	271.90	0.00000	H→L+3 (15.2%), H-1→L+2 (15.2%), H-4→L+2 (14.7%), H→L+4 (5.7%)
10	5.1914	238.83	0.00000	H-3→L+2 (38.2%), H-2→L+3 (38.2%)
11	5.7736	214.74	0.00000	H→L+1 (45.6%), H-1→L (45.6%)
12	6.1287	202.30	0.00000	H-1→L+5 (20.7%), H-1→L+4 (13.1%), H-4→L+3 (10.3%), H→L+5 (8.1%), H→L+2 (6.6%), H-1→L+3 (6.6%), H-1→L+9 (6.0%)
13	6.1287	202.30	0.00000	H→L+5 (20.7%), H→L+4 (13.1%), H-4→L+2 (10.3%), H-1→L+5 (8.1%), H-1→L+2 (6.6%), H→L+3 (6.6%), H→L+9 (6.0%)
14	6.1339	202.13	0.40410	H-1→L (17.8%), H→L+1 (17.8%), H-4→L+1 (16.9%), H-4→L (8.0%), H-5→L+3 (7.6%), H→L+6 (5.3%)
15	6.1339	202.13	0.40400	H-1→L+1 (17.8%), H→L (17.8%), H-4→L (16.9%), H-4→L+1 (8.0%), H-5→L+2 (7.6%), H-1→L+6 (5.3%)
16	6.1404	201.92	0.00080	H-3→L (29.4%), H-2→L+1 (29.4%), H-3→L+1 (12.1%), H-2→L (12.1%)
17	6.1719	200.88	0.00090	H→L+2 (28.8%), H-1→L+3 (28.8%), H-1→L+2 (8.0%), H→L+3 (8.0%), H-4→L+5 (5.1%)
18	6.2237	199.21	0.00000	H-5→L (28.1%), H-3→L (11.9%), H-2→L+1 (11.9%), H-5→L+1 (9.4%), H-3→L+10 (8.0%), H-7→L+1 (6.4%)
19	6.2237	199.21	0.00000	H-5→L+1 (28.1%), H-2→L (11.9%), H-3→L+1 (11.9%), H-5→L (9.4%), H-2→L+10 (8.0%), H-7→L (6.4%)
20	6.2941	196.98	0.00000	H-1→L+4 (36.2%), H-4→L+3 (9.7%), H→L+5 (6.3%), H-1→L+5 (5.4%), H-1→L+9 (5.3%)

Table S7 (c). Excited energy (in eV), excited wavelength (in nm), oscillator strength (*f*) and main molecular orbital transitions of 20 excited states of **C3** at gas phase

State	Energy/eV	λ/nm	*f*	Mayor MO transitions
1	1.9553	634.09	0.00000	H → L (98.6%)
**2**	**2.0467**	**605.78**	**0.00950**	**H-1 → L (50.1%), H → L+1 (48.9%)**
3	2.0576	602.57	0.00000	H-1 → L+1 (98.4%)

**4**	**2.2841**	**542.81**	**0.11350**	**H → L+1 (50.5%), H-1 → L (49.4%)**
5	3.2068	386.63	0.00000	H-2 → L (53.5%), H-5 → L (36.7%)
6	3.4221	362.30	0.00160	H-2 → L+1 (32.5%), H-3 → L (26.0%), H-5 → L+1 (18.0%), H-6 → L (16.4%)
7	3.5069	353.54	0.00000	H-3 → L+1 (54.9%), H-6 → L+1 (32.5%)
8	3.7990	326.36	0.00900	H → L+2 (77.6%)
9	3.8121	325.24	0.00000	H-1 → L+2 (82.5%), H-1 → L+9 (8.7%), H-6 → L+1 (6.2%)
10	4.0998	302.42	0.00000	H → L+3 (82.0%), H-5 → L (8.2%)
11	4.3085	287.77	0.00380	H-1 → L+3 (81.3%), H-3 → L (7.4%)
12	4.7635	260.28	0.00000	H-4 → L (40.0%), H-10 → L (20.9%), H-2 → L+3 (18.5%)
13	4.8947	253.30	0.00000	H → L+4 (64.7%), H → L+12 (9.6%), H → L+10 (5.8%)
14	4.9457	250.69	0.01000	H-4 → L+1 (28.1%), H-7 → L (19.4%), H-2 → L+2 (12.8%), H-11 → L (11.1%), H-10 → L+1 (9.6%), H-3 → L+3 (9.3%)
15	4.9563	250.15	0.13490	H-1 → L+4 (42.1%), H-2 → L+1 (17.3%), H-1 → L+12 (6.6%)
16	4.9934	248.30	0.00080	H-7 → L+1 (39.7%), H-3 → L+2 (22.1%), H-11 → L+1 (20.4%), H-4 → L (5.3%)
**17**	**5.0412**	**245.94**	**0.47950**	**H-2 → L+1 (33.2%), H-1 → L+4 (26.0%), H-1 → L+12 (10.0%), H → L+2 (9.4%), H-3 → L (8.9%)**
18	5.0791	244.11	0.00000	H-5 → L (33.4%), H-2 → L (27.6%), H → L+3 (11.5%), H → L+4 (6.2%)
19	5.1731	239.67	0.00000	H-6 → L+1 (41.1%), H-3 → L+1 (28.0%), H-7 → L+2 (6.7%), H-1 → L+9 (6.6%)
20	5.2300	237.06	0.19440	H-3 → L (43.0%), H-5 → L+1 (17.1%), H-6 → L (14.8%), H-1 → L+3 (9.4%)

Table S7 (d). Excited energy (in eV), excited wavelength (in nm), oscillator strength (*f*) and main molecular orbital transitions of 20 excited states of **C4** at gas phase

State	Energy/eV	λ/nm	*f*	Mayor MO transitions
1	2.8395	436.64	0.00000	H→L (93.4%)
2	2.9169	425.05	0.00500	H-1→L (54.6%); H→L+1 (40.4%)
3	2.9225	424.24	0.00010	H-1→L+1 (92.9%)
4	3.0685	404.05	0.00000	H-2→L (47.0%); H-4→L (44.9%)
5	3.2570	380.67	0.00000	H-2→L+1 (37.9%); H-4→L+1 (20.1%); H-5→L (18.5%); H-3→L (14.4%)
**6**	**3.3085**	**374.74**	**0.24640**	**H→L+1 (56.4%); H-1→L (42.6%)**
7	3.3592	369.09	0.00000	H-3→L+1 (45.3%); H-5→L+1 (39.8%); H-1→L+2 (8.5%)
8	3.7634	329.45	0.03090	H-2→L+1 (33.3%); H-3→L (25.0%); H→L+2 (18.1%); H-5→L (15.3%)
9	3.8124	325.21	0.00000	H-5→L+1 (33.3%); H-1→L+2 (32.0%); H-3→L+1 (9.9%); H-1→L+5 (7.7%)
10	3.9254	315.85	0.00000	H-4→L (40.3%); H-2→L (28.4%); H→L+3 (12.0%)
11	3.9575	313.29	0.01140	H-3→L (42.8%); H-4→L+1 (15.4%); H-5→L (14.3%); H-1→L+3 (11.9%); H→L+2 (6.0%)

12	4.2354	292.73	0.00000	H-3→L+1 (33.0%); H-1→L+5 (32.1%); H-1→L+2 (15.8%); H-5→L+1 (13.7%)
13	4.3301	286.33	0.01270	H-1→L+4 (24.3%); H→L+5 (19.1%); H→L+2 (17.3%); H-2→L+1 (14.3%); H-3→L (5.5%)
14	4.4308	279.82	0.00000	H→L+4 (60.1%); H→L+3 (17.3%); H-2→L (6.8%); H→L+7 (5.4%)
15	4.7982	258.40	0.00070	H-3→L+2 (53.9%); H-3→L+5 (27.7%)
16	4.9046	252.79	0.01340	H-2→L+2 (39.9%); H-3→L+4 (15.7%); H-2→L+5 (13.1%); H-3→L+3 (10.1%)
17	4.9646	249.74	0.00020	H-2→L+3 (30.4%); H-6→L (16.9%); H-2→L+4 (16.6%); H-4→L+3 (8.9%); H-11→L (5.0%)
18	5.0245	246.76	0.01320	H→L+2 (18.8%); H-4→L+1 (18.6%); H-1→L+3 (16.2%); H-1→L+4 (14.8%); H-5→L (11.2%); H-2→L+1 (9.8%); H-3→L (5.4%)
19	5.0863	243.76	0.00010	H-2→L+4 (31.1%); H-6→L (15.9%); H-4→L+3 (11.1%); H-11→L (7.9%); H-7→L+1 (7.9%)
20	5.3007	233.90	0.00020	H-7→L+1 (34.6%); H-5→L+2 (21.2%); H-10→L+1 (11.5%); H-6→L (7.7%); H-3→L+5 (5.5%)

Table S7 (e). Excited energy (in eV), excited wavelength (in nm), oscillator strength (*f*) and main molecular orbital transitions of 20 excited states of **C5** at gas phase

State	Energy/eV	λ/nm	*f*	Mayor MO transitions
1	3.2167	385.44	0.00000	H-1→L (47.2%); H→L+1 (47.2%)
2	3.2962	376.14	0.00000	H-3→L+1 (25.2%); H-2→L (25.2%); H→L+3 (22.1%); H-1→L+2 (22.1%)
3	3.3517	369.91	0.00000	H-2→L+2 (46.5%); H-3→L+3 (46.5%)
4	3.4679	357.52	0.00000	H→L (34.1%); H-1→L+1 (34.1%); H-4→L+1 (19.5%)
5	3.4679	357.52	0.00000	H-1→L (34.1%); H→L+1 (34.1%); H-4→L (19.5%)
6	3.5667	347.62	0.00080	H-1→L+3 (18.1%); H→L+2 (18.1%); H-2→L+1 (17.9%); H-3→L (17.9%); H-4→L+3 (9.9%); H-5→L+1 (9.6%)
7	3.5669	347.60	0.00080	H-1→L+2 (18.1%); H→L+3 (18.1%); H-2→L (17.9%); H-3→L+1 (17.9%); H-4→L+2 (9.9%); H-5→L (9.6%)
8	3.6026	344.15	0.00000	H-3→L+3 (33.7%); H-2→L+2 (33.7%); H-5→L+2 (18.2%)
9	3.6027	344.14	0.00000	H-3→L+2 (33.7%); H-2→L+3 (33.7%); H-5→L+3 (18.2%)
10	3.9385	314.80	0.00000	H→L+3 (26.1%); H-1→L+2 (26.1%); H-2→L (23.2%); H-3→L+1 (23.2%)
11	4.2880	289.14	0.00040	H→L (35.6%); H-1→L+1 (35.6%); H-4→L+5 (13.0%)
12	4.3617	284.26	0.00000	H-3→L (21.1%); H-2→L+1 (21.1%); H→L+2 (19.0%); H-1→L+3 (19.0%); H-4→L+4 (8.8%); H-5→L+5 (7.2%)
13	4.3755	283.36	0.00070	H-2→L+3 (33.2%); H-3→L+2 (33.2%); H-5→L+4 (16.0%); H-1→L+1 (5.5%); H→L (5.5%)
14	4.4717	277.26	0.00000	H-2→L+4 (24.7%); H-5→L+3 (22.4%); H-4→L+1 (19.8%); H-1→L+5 (15.2%)
15	4.4717	277.26	0.00000	H-3→L+4 (24.7%); H-5→L+2 (22.4%); H-4→L (19.8%); H→L+5

				(15.2%)
16	4.5655	271.57	0.00010	H-1→L+4 (23.2%); H-4→L+3 (22.5%); H-5→L+1 (22.0%); H-2→ L+5 (16.4%)
17	4.5655	271.57	0.00010	H→L+4 (23.1%); H-4→L+2 (22.5%); H-5→L (22.0%); H-3→L+5 (16.4%)
18	4.5668	271.49	0.00000	H-4→L+1 (28.0%); H-1→L+5 (20.2%); H-2→L+4 (18.3%); H-5→ L+3 (17.4%)
19	4.5668	271.49	0.00000	H-4→L (28.0%); H→L+5 (20.2%); H-3→L+4 (18.3%); H-5→L+2 (17.4%)
20	4.9057	252.73	0.00000	H-1→L+3 (24.1%); H→L+2 (24.1%); H-2→L+1 (22.9%); H-3→L (22.9%)

Table S7 (f). Excited energy (in eV), excited wavelength (in nm), oscillator strength (*f*) and main molecular orbital transitions of 20 excited states of **C6** at gas phase

State	Energy/eV	λ/nm	*f*	Mayor MO transitions
1	3.2793	378.08	0.00000	H-2→L (62.3%); H→L+2 (19.2%); H-3→L+1 (9.0%)
2	3.3277	372.58	0.00190	H-3→L (39.9%); H-2→L+1 (36.6%); H-1→L+2 (9.0%); H→L+3 (6.2%)
3	3.3335	371.93	0.00000	H-3→L+1 (72.7%); H-1→L+3 (8.3%); H-2→L (6.1%)
4	3.4141	363.15	0.00000	H→L (52.0%); H-1→L+1 (23.2%); H-4→L+2 (6.2%)
5	3.4718	357.12	0.00000	H→L+2 (51.9%); H-2→L (21.9%); H-4→L (10.8%); H→L+6 (7.8%)
6	3.4779	356.49	0.00000	H-1→L+1 (50.5%); H→L (25.4%); H-5→L+3 (6.0%)
7	3.4817	356.10	0.00000	H→L+1 (38.5%); H-1→L (38.3%)
8	3.5778	346.54	0.00000	H-1→L+2 (38.6%); H→L+3 (20.3%); H-2→L+1 (11.6%); H-5→L (6.9%); H-4→L+1 (5.7%)
9	3.6379	340.81	0.00000	H-1→L+3 (58.8%); H-5→L+1 (16.1%); H-3→L+1 (7.7%); H-1→ L+5 (5.3%)
10	3.9712	312.21	0.00210	H→L+3 (34.1%); H-3→L (23.2%); H-2→L+1 (19.8%); H-1→L+2 (16.0%)
11	4.2801	289.68	0.00000	H-3→L+3 (55.5%); H-2→L+2 (23.5%)
**12**	**4.3164**	**287.24**	**0.32840**	**H-1→L (51.9%); H→L+1 (39.5%); H-3→L+2 (6.1%)**
13	4.3563	284.61	0.01860	H-2→L+3 (43.4%); H-3→L+2 (33.2%); H→L+1 (11.0%)
14	4.3581	284.49	0.00040	H-2→L+2 (58.2%); H-3→L+3 (23.3%)
15	4.6781	265.03	0.00000	H-4→L (33.4%); H-7→L+2 (20.2%); H→L+6 (13.7%); H-5→L+1 (8.5%); H-6→L+3 (5.4%)
16	4.7718	259.83	0.02490	H-4→L+1 (21.3%); H-5→L (20.1%); H-6→L+2 (12.9%); H-7→ L+3 (10.8%); H-1→L+6 (9.5%); H→L+5 (6.7%); H→L+7 (6.0%)
17	4.7862	259.05	0.00000	H-5→L+1 (28.3%); H-6→L+3 (16.3%); H-4→L (11.6%); H-1→ L+5 (10.8%); H-1→L+7 (9.4%); H-7→L+2 (6.5%)
18	5.0303	246.47	0.00000	H-4→L+2 (30.8%); H-7→L (17.4%); H-5→L+3 (13.9%); H-6→ L+1 (9.0%); H→L (8.4%); H-1→L+1 (5.8%)
19	5.1214	242.09	0.00330	H-5→L+2 (23.3%); H-4→L+3 (22.2%); H-6→L (13.9%); H-7→

				L+1 (13.2%); H→L+1 (7.7%); H-1→L (5.5%)
20	5.1226	242.03	0.00020	H-5→L+3 (27.7%); H-6→L+1 (17.7%); H-4→L+2 (17.2%); H-1→L+1 (9.8%); H-7→L (9.3%)

Table S7 (g). Excited energy (in eV), excited wavelength (in nm), oscillator strength (*f*) and main molecular orbital transitions of 20 excited states of **C7** at gas phase

State	Energy/eV	λ/nm	*f*	Mayor MO transitions
1	3.3674	368.19	0.00000	H-2→L (43.7%); H-3→L+1 (19.5%); H-4→L (11.1%); H→L+2 (9.1%); H-1→L+3 (6.0%)
2	3.3949	365.21	0.00000	H→L+1 (54.0%); H-1→L (18.8%); H-3→L+2 (7.3%); H-6→L+1 (7.3%)
3	3.4059	364.03	0.00000	H-3→L+1 (34.9%); H-2→L (23.3%); H→L+2 (20.9%); H-5→L+1 (5.6%); H-4→L (5.6%)
4	3.4169	362.86	0.00010	H-2→L+1 (35.4%); H-3→L (26.9%); H-1→L+2 (11.6%); H→L+3 (7.3%); H-4→L+1 (6.4%); H-5→L (6.0%)
5	3.4550	358.85	0.00000	H-1→L (55.0%); H→L+1 (19.5%); H-7→L (9.3%)
6	3.4593	358.41	0.00010	H→L (38.9%); H-1→L+1 (36.0%); H-6→L (5.4%)
7	3.8117	325.27	0.00000	H→L+2 (51.3%); H-3→L+1 (16.2%); H-5→L+1 (10.1%); H-1→L+3 (7.6%)
8	3.8977	318.10	0.00050	H-1→L+2 (39.0%); H→L+3 (27.0%); H-3→L (8.7%); H-5→L (5.3%)
9	3.9331	315.23	0.00000	H-1→L+3 (67.0%); H→L+2 (5.6%)
**10**	**4.0305**	**307.61**	**0.02390**	**H-3→L (31.8%); H-2→L+1 (30.2%); H→L+3 (22.9%); H-1→L+2 (8.5%)**
**11**	**4.3676**	**283.87**	**0.43670**	**H-1→L+1 (48.9%); H→L (48.1%)**
12	4.3849	282.75	0.00010	H-3→L+2 (63.4%); H-2→L+3 (6.0%); H→L+8 (6.0%); H→L+1 (5.9%); H-5→L+2 (5.2%)
13	4.4686	277.46	0.00010	H-2→L+2 (38.4%); H-3→L+3 (32.1%)
14	4.4950	275.83	0.00020	H-2→L+3 (62.6%); H-1→L+6 (7.6%); H-3→L+2 (6.7%); H-4→L+3 (5.5%)
15	4.8766	254.24	0.00000	H-7→L (30.6%); H-6→L+1 (27.5%); H-1→L (10.0%); H→L+1 (6.5%)
16	4.9716	249.38	0.00000	H-6→L+1 (36.2%); H-7→L (27.0%); H→L+1 (7.1%); H-1→L (7.0%)
17	4.9729	249.32	0.00010	H-6→L (32.5%); H-7→L+1 (30.6%); H→L (7.4%); H-1→L+1 (6.8%)
18	4.9755	249.19	0.00000	H-4→L (30.3%); H-5→L+1 (24.0%); H-1→L+4 (7.0%); H→L+5 (5.5%); H-2→L (5.5%)
19	5.0770	244.21	0.00430	H-5→L (29.7%); H-4→L+1 (29.0%); H-3→L (7.6%); H-1→L+5 (5.8%); H→L+4 (5.2%)
20	5.0771	244.20	0.00000	H-5→L+1 (36.9%); H-4→L (22.7%); H-3→L+1 (7.1%); H→L+5 (6.7%)

Table S7 (h). Excited energy (in eV), excited wavelength (in nm), oscillator strength (*f*) and main molecular orbital transitions of 20 excited states of **C8** at gas phase

State	Energy/eV	λ/nm	*f*	Mayor MO transitions
1	3.5240	351.83	0.00000	H→L+1 (45.8%); H-1→L (45.8%)
2	3.5915	345.22	0.00000	H-3→L (24.8%); H-2→L+1 (24.8%); H-1→L+3 (22.2%); H→L+2 (22.2%)
3	3.6253	342.00	0.00000	H-3→L+3 (45.2%); H-2→L+2 (45.2%)
4	3.8377	323.07	0.00000	H-1→L (33.1%); H→L+1 (33.1%); H-4→L+1 (12.1%)
5	3.8378	323.06	0.00000	H-1→L+1 (33.1%); H→L (33.1%); H-4→L (12.1%)
6	3.9197	316.31	0.00050	H→L+3 (18.6%); H-1→L+2 (18.6%); H-2→L (18.4%); H-3→L+1 (18.4%); H-4→L+3 (6.9%); H-5→L (6.8%)
7	3.9198	316.30	0.00050	H→L+2 (18.6%); H-1→L+3 (18.6%); H-2→L+1 (18.4%); H-3→L (18.4%); H-4→L+2 (6.9%); H-5→L+1 (6.8%)
8	3.9368	314.94	0.00000	H-2→L+2 (32.6%); H-3→L+3 (32.5%); H-5→L+2 (10.9%)
9	3.9368	314.94	0.00000	H-3→L+2 (32.5%); H-2→L+3 (32.5%); H-5→L+3 (10.9%)
10	4.2464	291.97	0.00000	H→L+2 (25.8%); H-1→L+3 (25.8%); H-3→L (23.3%); H-2→L+1 (23.3%)
11	4.7978	258.42	0.00000	H-1→L+1 (24.3%); H→L (24.3%); H-3→L+2 (15.6%); H-2→L+3 (15.6%); H-4→L+5 (8.2%); H-5→L+4 (7.5%)
12	4.8002	258.29	0.00000	H-5→L+3 (29.4%); H-2→L+4 (25.7%); H-4→L (14.1%); H→L+5 (9.7%)
13	4.8002	258.29	0.00000	H-5→L+2 (29.4%); H-3→L+4 (25.7%); H-4→L+1 (14.1%); H-1 →L+5 (9.7%)
14	4.8645	254.88	0.00030	H-2→L+3 (23.0%); H-3→L+2 (23.0%); H→L (17.8%); H-1→L+1 (17.8%); H-5→L+4 (9.5%); H-4→L+5 (5.1%)
15	4.8656	254.82	0.00000	H-3→L+1 (21.8%); H-2→L (21.8%); H-1→L+2 (19.4%); H→L+3 (19.4%); H-4→L+4 (7.7%); H-5→L+5 (6.6%)
16	4.9433	250.81	0.00010	H-4→L+3 (22.7%); H-5→L (22.6%); H→L+4 (20.5%); H-2→L+5 (15.5%)
17	4.9434	250.81	0.00010	H-4→L+2 (22.7%); H-5→L+1 (22.6%); H-1→L+4 (20.5%); H-3 →L+5 (15.5%)
18	4.9503	250.46	0.00000	H-4→L+1 (33.6%); H-1→L+5 (22.8%); H-5→L+2 (12.9%); H-3 →L+4 (11.7%)
19	4.9503	250.46	0.00000	H-4→L (33.6%); H→L+5 (22.8%); H-5→L+3 (12.9%); H-2→L+4 (11.7%)
20	5.3227	232.93	0.00000	H→L+3 (24.5%); H-1→L+2 (24.5%); H-2→L (22.9%); H-3→L+1 (22.9%)

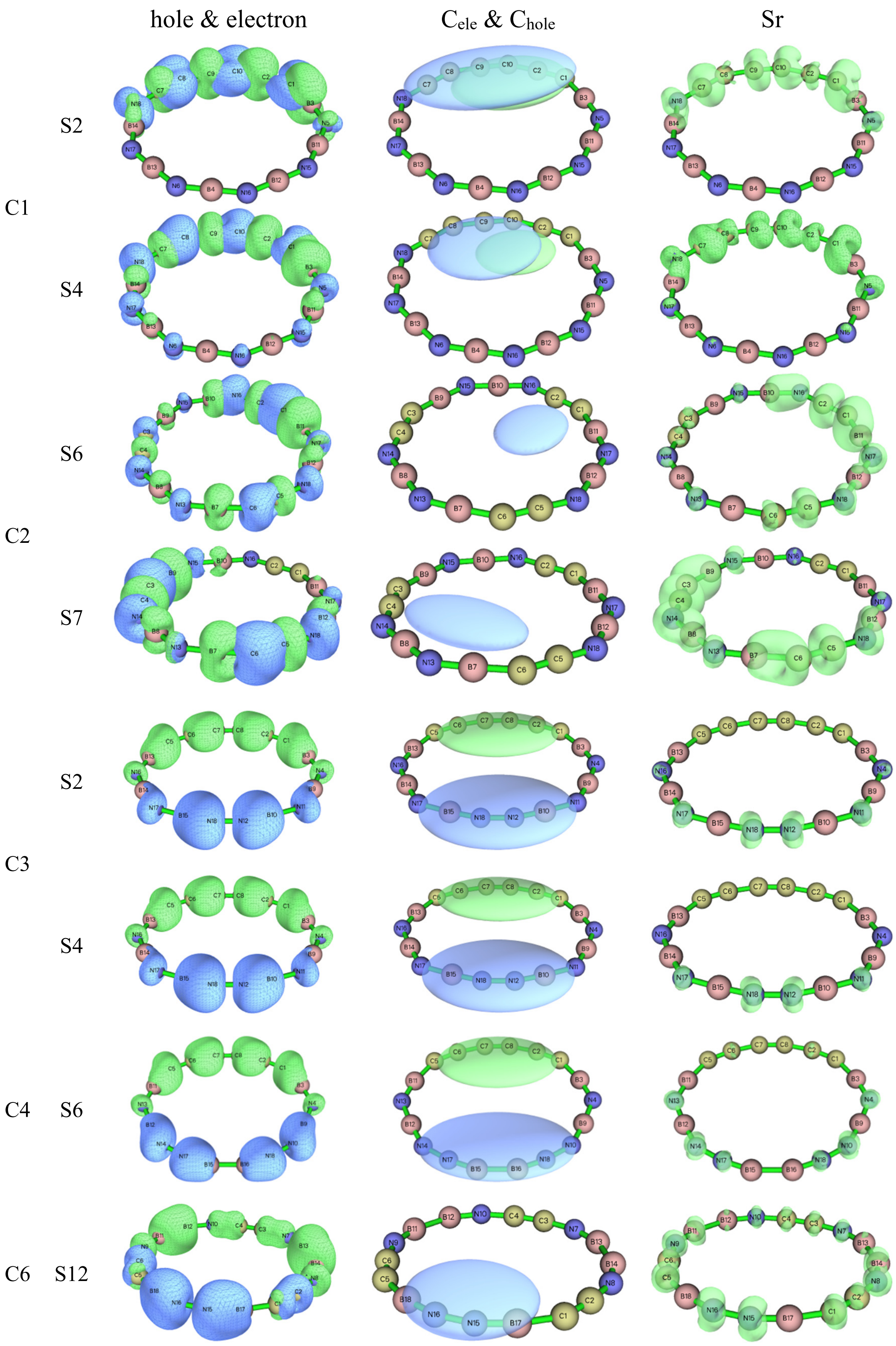
hole & electron
Cele & Chole
Sr
S2
C1
S4
S6
C2
S7
S2
C3
S4
C4
S6
C6
S12

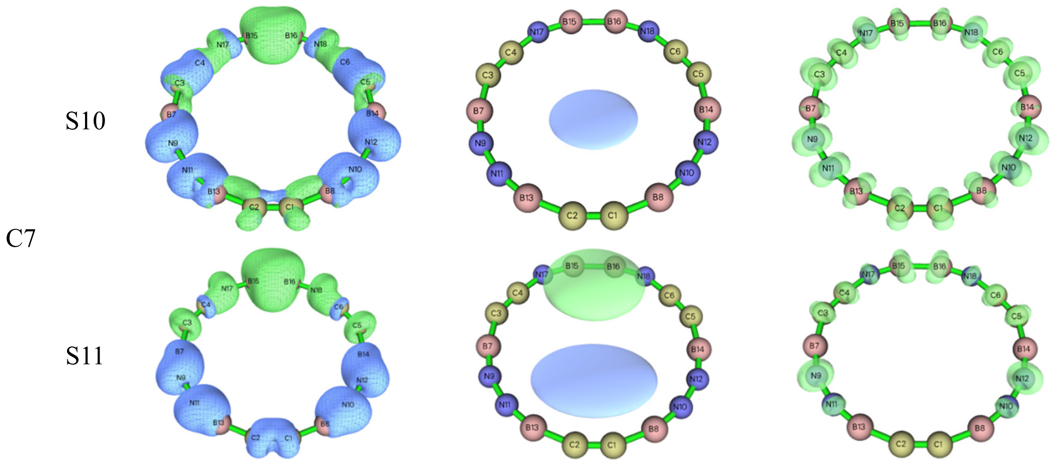

Figure S7. The isosurface of hole and electron distribution, isosurface of C_{ele} and C_{hole} functions and isosurface of overlap of hole-electron (Sr) of various excitation. (Isovalue = 0.002 a.u..)

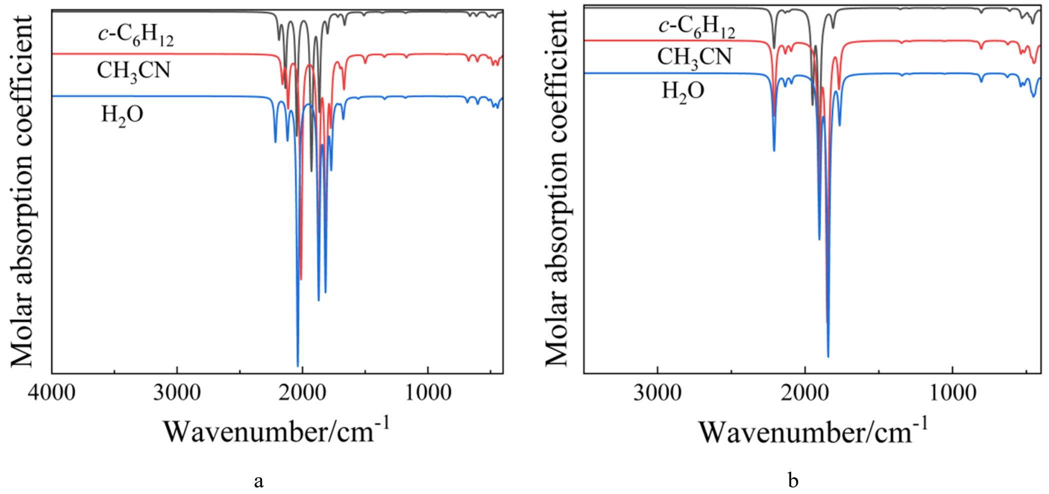

Figure S8. Simulated IR spectra of compound C1 (a) and C3 (b) in cyclo-hexane, acetonitrile and water

Table S8 (a). Excited energy (in eV), excited wavelength (in nm), oscillator strength (*f*) and main molecular orbital transitions of 20 excited states of **C1** in cyclohexane

State	Energy/eV	λ/nm	*f*	Mayor MO transitions
1	2.8223	439.30	0.0001	H→L+1 84.8%, H-1→L 9.0%
**2**	**2.9258**	**423.76**	**0.0235**	**H→L 58.7%, H-1→L+1 35.2%**
3	2.9807	415.96	0.0007	H-1→L 84.7%, H→L+1 9.1%
**4**	**4.3743**	**283.44**	**1.52650**	**H-1→L+1 56.4%, H→L 33.3%**
5	4.8593	255.15	0.0000	H-4→L+1 21.2%, H-2→L+1 14.5%, H-5→L 14.4%, H-3→L 9.1%, H-1→L+2 8.7%, H→L+6 8.6%, H→L+3 6.5%, H-1→L+7 5.3%
6	4.9657	249.68	0.0906	H→L+2 19.2%, H-2→L 16.5%, H-4→L 16.3%, H-5→L+1 13.7%, H-3→L+1 8.0%, H→L+7 6.9%, H-1→L+6 5.6%
7	4.9954	248.20	0.0000	H-5→L 19.7%, H-1→L+2 15.0%, H-3→L 14.1%, H-4→L+1 13.7%,

				H-2→L+1 10.3%, H-1→L+7 7.6%, H→L+6 5.6%
8	5.6459	219.60	0.0845	H→L+2 49.6%, H-2→L 22.8%
9	5.8588	211.62	0.0003	H→L+4 42.3%, H→L+3 18.6%, H→L+10 10.4%, H→L+5 9.1%, H→L+8 5.8%
10	5.8967	210.26	0.0000	H-1→L+2 35.0%, H-3→L 13.4%, H→L+4 7.5%, H→L+3 6.5%, H-5→L 6.0%
11	5.9391	208.76	0.0051	H→L+3 19.9%, H-2→L+1 13.7%, H-1→L+2 13.5%, H→L+4 9.8%, H-3→L 6.9%, H→L+8 5.8%
12	6.0858	203.73	0.6634	H-2→L+2 17.9%, H-2→L 13.1%, H-4→L 13.0%, H→L+2 11.6%, H-5→L+1 9.1%, H-6→L 8.2%
13	6.1067	203.03	0.0002	H-3→L+2 32.3%, H-5→L 17.7%, H-7→L 17.0%, H-1→L+2 7.2%, H-9→L 5.1%
14	6.2475	198.45	0.0009	H→L+6 26.5%, H-2→L+3 21.1%, H→L+8 10.4%, H-6→L+1 5.5%
15	6.2547	198.23	0.6504	H-2→L+2 25.7%, H-2→L 23.8%, H-1→L+6 6.3%, H-3→L+3 5.8%, H-1→L+4 5.7%
16	6.3450	195.40	0.0728	H-1→L+4 59.9%, H-1→L+10 13.9%, H-1→L+3 7.9%
17	6.3525	195.17	0.0000	H-2→L+1 14.9%, H-6→L+1 11.7%, H-4→L+1 11.2%, H→L+3 6.5%, H-8→L+1 6.5%, H-2→L+3 6.3%, H-6→L+3 5.5%
18	6.4531	192.13	0.0002	H-3→L+2 26.2%, H-3→L 23.2%, H-7→L+2 10.7%, H-5→L 9.4%, H-9→L 5.3%, H-3→L+9 5.0%
19	6.5652	188.85	0.0000	H-2→L+3 22.7%, H-4→L+3 10.1%, H-2→L+6 8.5%, H-10→L+1 6.5%, H-11→L 6.1%, H-5→L+2 5.2%
20	6.5957	187.98	0.0782	H-1→L+3 25.9%, H-3→L+1 12.0%, H-1→L+8 9.9%, H-7→L+1 5.9%

Table S8 (b). Excited energy (in eV), excited wavelength (in nm), oscillator strength (*f*) and main molecular orbital transitions of 20 excited states of **C1** in CH_3CN

State	Energy/eV	λ/nm	*f*	Mayor MO transitions
1	2.9035	427.02	0.0001	H→L+1 87.7%, H-1→L 5.7%
**2**	**3.0300**	**409.19**	**0.0235**	**H→L 58.6%, H-1→L+1 35.2%**
3	3.1059	399.19	0.0007	H-1→L 88.1%, H→L+1 5.8%
**4**	**4.5088**	**274.98**	**1.4606**	**H-1→L+1 55.3%, H→L 33.0%**
5	4.9066	252.69	0.0001	H-4→L+1 29.7%, H-2→L+1 17.0%, H→L+3 10.8%, H→L+5 9.5%, H-5→L 7.6%, H→L+6 6.0%
6	5.0524	245.40	0.0928	H-4→L 17.7%, H→L+2 16.4%, H-2→L 16.4%, H-5→L+1 15.7%, H→L+7 6.7%, H-3→L+1 6.1%, H-1→L+3 5.2%
7	5.0943	243.38	0.0000	H-5→L 32.9%, H-1→L+2 18.6%, H-3→L 15.5%, H-1→L+7 10.2%, H-4→L+1 6.1%
8	5.7569	215.37	0.1032	H→L+2 43.5%, H-2→L 27.9%, H-2→L+2 5.9%
9	5.9681	207.74	0.0032	H-2→L+1 22.8%, H→L+4 17.2%, H→L+5 14.4%, H→L+3 14.0%, H→L+10 5.3%
10	6.0237	205.83	0.0000	H→L+4 31.4%, H→L+3 21.0%, H→L+8 10.2%, H→L+10 9.1%, H→L+6 6.4%

11	6.0771	204.02	0.0025	H-1→L+2 33.9%, H-3→L 23.1%, H-3→L+2 12.0%, H-7→L 9.4%
12	6.1476	201.68	0.6585	H→L+2 19.4%, H-2→L 17.0%, H-4→L 15.3%, H-5→L+1 11.3%, H-2→L+2 7.2%
13	6.2116	199.60	0.0005	H-5→L 22.7%, H-1→L+2 21.0%, H-3→L+2 20.8%, H-7→L 11.6%, H-9→L 5.3%
14	6.3012	196.76	0.6848	H-2→L+2 38.7%, H-2→L 18.1%, H-3→L+3 7.4%
15	6.3430	195.47	0.0006	H-2→L+3 21.4%, H→L+8 13.0%, H→L+6 12.0%, H→L+5 9.9%, H-6→L+1 5.8%, H→L+4 5.8%
16	6.3936	193.92	0.0002	H-4→L+1 18.0%, H-6→L+1 12.1%, H→L+3 12.0%, H-2→L+1 10.3%, H-8→L+1 8.3%
17	6.5065	190.55	0.0001	H-3→L+2 31.1%, H-3→L 25.1%, H-7→L+2 9.6%, H-5→L 6.7%
18	6.5558	189.12	0.0118	H-1→L+4 59.3%, H-1→L+10 18.4%, H-1→L+5 5.1%
19	6.6146	187.44	0.0000	H-2→L+3 20.2%, H-4→L+3 9.0%, H→L+14 7.4%, H-2→L+5 7.1%, H-10→L+1 6.3%
20	6.6570	186.25	0.0456	H-6→L 13.8%, H-7→L+1 10.4%, H-3→L+1 10.1%, H-10→L 6.4%, H-11→L+1 6.1%, H-4→L+2 5.4%

Table S8 (c). Excited energy (in eV), excited wavelength (in nm), oscillator strength (*f*) and main molecular orbital transitions of 20 excited states of **C1** in H_2O

State	Energy/eV	λ/nm	*f*	Mayor MO transitions
1	2.9070	426.50	0.0002	H→L+1 88.3%
**2**	**3.0422**	**407.55**	**0.0230**	**H→L 58.3%, H-1→L+1 35.3%**
3	3.1248	396.77	0.0006	H-1→L 88.7%, H→L+1 5.1%
**4**	**4.5282**	**273.80**	**1.4518**	**H-1→L+1 54.9%, H→L 33.0%, H-2→L+2 5.1%**
5	4.9198	252.01	0.0000	H-4→L+1 30.7%, H-2→L+1 17.9%, H→L+5 10.1%, H→L+3 7.6%, H-5→L 7.2%, H→L+6 5.7%
6	5.0704	244.53	0.0987	H-4→L 17.7%, H-2→L 17.0%, H-5→L+1 16.2%, H→L+2 15.6%, H →L+7 6.5%, H-3→L+1 5.9%
7	5.1158	242.36	0.0000	H-5→L 34.4%, H-1→L+2 18.3%, H-3→L 15.1%, H-1→L+7 10.2%, H-4→L+1 5.8%
8	5.7691	214.91	0.1136	H→L+2 43.6%, H-2→L 28.0%, H-2→L+2 6.2%
9	5.9820	207.26	0.00270	H→L+4 36.5%, H→L+5 19.7%, H-2→L+1 13.7%, H→L+10 10.5%
10	6.0252	205.78	0.0007	H→L+3 41.2%, H-2→L+1 12.9%, H→L+8 12.6%, H→L+6 6.7%
11	6.1003	203.24	0.0023	H-1→L+2 31.0%, H-3→L 23.4%, H-3→L+2 14.2%, H-7→L 10.6%
12	6.1512	201.56	0.6424	H→L+2 20.2%, H-2→L 18.0%, H-4→L 15.6%, H-5→L+1 11.5%, H-2→L+2 5.6%
13	6.2293	199.03	0.0006	H-1→L+2 24.6%, H-5→L 22.7%, H-3→L+2 18.6%, H-7→L 10.4%, H-9→L 5.1%
14	6.3041	196.67	0.6630	H-2→L+2 40.1%, H-2→L 16.7%, H-3→L+3 7.8%
15	6.3408	195.53	0.0008	H-2→L+3 16.2%, H→L+6 15.4%, H→L+4 11.7%, H→L+8 10.4%, H→L+5 10.1%, H-2→L+1 6.6%
16	6.3889	194.06	0.0005	H-4→L+1 20.2%, H-6→L+1 14.7%, H-8→L+1 8.2%, H→L+3 7.6%, H-2→L+1 7.0%, H→L+4 6.2%, H-2→L+3 5.4%

17	6.5055	190.58	0.0001	H-3→L+2 32.0%, H-3→L 25.5%, H-7→L+2 9.3%, H-5→L 6.2%
18	6.5715	188.67	0.0161	H-1→L+4 53.8%, H-1→L+10 17.8%, H-1→L+5 5.9%
19	6.6254	187.13	0.0000	H-2→L+3 21.0%, H-4→L+3 8.7%, H-10→L+1 6.6%, H-2→L+5 6.4%, H→L+14 6.0%
20	6.6678	185.94	0.0645	H-6→L 15.6%, H-7→L+1 10.6%, H-3→L+1 9.2%, H-10→L 6.8%, H-11→L+1 6.2%, H-4→L+2 5.7%, H-2→L+2 5.4%, H-2→L+7 5.3%

Table S8 (d). Excited energy (in eV), excited wavelength (in nm), oscillator strength (*f*) and main molecular orbital transitions of 20 excited states of **C3** in cyclohexane

State	Energy/eV	λ/nm	*f*	Mayor MO transitions
1	2.4385	508.44	0.0000	H→L 98.5%
**2**	**2.5188**	**492.24**	**0.0393**	**H→L+1 72.7%, H-1→L 26.1%**
3	2.5631	483.73	0.0001	H-1→L+1 98.4%
4	2.6794	462.73	0.1023	H-1→L 73.3%, H→L+1 26.4%
5	3.2367	383.06	0.0000	H-2→L 62.8%, H-4→L 27.5%
6	3.4425	360.16	0.0013	H-2→L+1 35.7%, H-3→L 35.6%, H-4→L+1 13.8%, H-5→L 10.0%
7	3.5199	352.24	0.0000	H-3→L+1 70.4%, H-5→L+1 19.3%
8	4.1063	301.94	0.0018	H→L+2 73.3%, H-2→L+1 7.2%, H→L+9 5.8%, H-3→L 5.5%
9	4.1615	297.93	0.0000	H-1→L+2 80.9%, H-1→L+9 12.1%
10	4.4845	276.47	0.0000	H→L+3 73.8%, H-4→L 10.9%
11	4.6785	265.01	0.1633	H-1→L+3 60.3%, H-3→L 14.6%, H-2→L+1 9.8%
12	4.8663	254.78	0.0000	H-6→L 32.2%, H-8→L 23.8%, H-2→L+3 21.0%
13	4.9916	248.39	0.0000	H→L+4 65.6%, H→L+11 8.8%, H→L+10 5.6%
14	5.0506	245.48	0.0149	H-6→L+1 23.2%, H-7→L 15.5%, H-2→L+2 15.1%, H-9→L 13.6%, H-8→L+1 11.9%, H-3→L+3 11.3%
**15**	**5.0843**	**243.86**	**1.0670**	**H-2→L+1 29.0%, H-1→L+3 23.9%, H→L+2 12.1%, H-3→L 9.9%, H-5→L 7.6%, H-1→L+4 6.6%**
16	5.0942	243.38	0.0011	H-7→L+1 30.3%, H-3→L+2 25.5%, H-9→L+1 25.0%, H-6→L 5.5%
17	5.1029	242.97	0.4395	H-1→L+4 57.5%, H-1→L+11 14.4%, H-3→L 7.5%, H-2→L+1 6.0%
18	5.3500	231.75	0.0000	H-4→L 36.1%, H-2→L 18.9%, H→L+3 14.9%
19	5.4312	228.28	0.0000	H-5→L+1 48.0%, H-3→L+1 14.0%, H-1→L+9 10.8%, H-7→L+2 7.9%
20	5.4536	227.34	0.1880	H-4→L+1 31.0%, H-3→L 18.2%, H-5→L 14.1%, H-1→L+3 8.8%, H→L+9 5.6%

Table S8 (e). Excited energy (in eV), excited wavelength (in nm), oscillator strength (*f*) and main molecular orbital transitions of 20 excited states of **C3** in CH_3CN

State	Energy/eV	λ/nm	*f*	Mayor MO transitions
1	3.1843	389.36	0.0000	H→L 97.9%

**2**	**3.2548**	**380.93**	**0.0731**	**H→L+1 91.9%, H-1→L 6.5%**
3	3.2718	378.95	0.0000	H-2→L 76.5%, H-4→L 12.8%, H-3→L+1 6.1%
4	3.3752	367.34	0.0001	H-1→L+1 98.0%
5	3.4613	358.20	0.0473	H-1→L 92.7%, H→L+1 6.8%
6	3.4716	357.14	0.0007	H-3→L 43.8%, H-2→L+1 42.8%, H-4→L+1 6.4%
7	3.5372	350.52	0.0000	H-3→L+1 83.9%, H-2→L 5.8%, H-5→L+1 5.0%
8	4.5237	274.08	0.0443	H→L+2 56.2%, H-2→L+1 15.0%, H-3→L 13.0%, H→L+8 7.9%
9	4.7017	263.70	0.0000	H-1→L+2 73.6%, H-1→L+8 19.3%
10	4.9557	250.19	0.0000	H→L+4 44.2%, H→L+3 28.5%, H→L+9 11.1%, H-4→L 5.8%
11	4.9752	249.20	0.0000	H-8→L 25.0%, H-6→L 24.3%, H-2→L+3 23.3%, H-9→L+1 6.3%, H-3→L+2 5.7%
**12**	**5.0565**	**245.20**	**0.5686**	**H-1→L+4 27.7%, H-3→L 16.5%, H-2→L+1 16.0%, H-1→L+3 13.1%, H-1→L+9 8.7%, H→L+2 7.0%**
13	5.1689	239.87	0.0116	H-6→L+1 17.7%, H-2→L+2 17.6%, H-9→L 15.7%, H-3→L+3 13.9%, H-8→L+1 13.8%, H-7→L 13.2%
14	5.1788	239.41	0.0000	H→L+4 30.8%, H→L+3 29.1%, H-4→L 19.8%
15	5.1965	238.59	0.0009	H-3→L+2 27.2%, H-9→L+1 26.1%, H-7→L+1 22.4%, H-6→L 6.7%, H-2→L+3 5.2%
16	5.2953	234.14	0.8485	H-1→L+4 46.2%, H-3→L 12.4%, H→L+2 10.6%, H-2→L+1 10.4%, H-1→L+10 5.9%, H-1→L+11 5.8%
17	5.6038	221.25	0.3703	H-1→L+3 48.3%, H-5→L 14.5%, H-4→L+1 8.9%, H→L+2 8.3%, H-2→L+1 7.3%
18	5.7429	215.89	0.0000	H-4→L 24.6%, H→L+11 19.2%, H→L+3 10.9%, H→L+10 7.7%, H →L+14 5.1%, H-2→L 5.0%
19	5.8285	212.72	0.0000	H-5→L+1 49.8%, H-1→L+8 16.0%, H-7→L+2 10.1%, H-11→L+1 6.4%
20	5.8388	212.35	0.3867	H-4→L+1 27.3%, H-1→L+3 19.3%, H→L+8 12.4%, H-3→L 7.3%, H-1→L+11 6.1%

Table S8 (f). Excited energy (in eV), excited wavelength (in nm), oscillator strength (*f*) and main molecular orbital transitions of 20 excited states of **C3** in H_2O

State	Energy/eV	λ/nm	*f*	Mayor MO transitions
1	3.2101	386.23	0.0000	H→L 97.8%
2	3.2713	379.01	0.0000	H-2→L 77.0%, H-4→L 12.4%, H-3→L+1 6.0%
**3**	**3.2817**	**377.80**	**0.0737**	**H→L+1 92.5%, H-1→L 6.0%**
4	3.4061	364.01	0.0001	H-1→L+1 98.0%
5	3.4722	357.08	0.0007	H-3→L 44.0%, H-2→L+1 43.1%, H-4→L+1 6.2%
6	3.4886	355.40	0.0433	H-1→L 93.3%, H→L+1 6.2%
7	3.5387	350.37	0.0000	H-3→L+1 84.4%, H-2→L 5.8%
8	4.5408	273.04	0.0512	H→L+2 55.2%, H-2→L+1 15.5%, H-3→L 13.5%, H→L+8 7.9%
9	4.7283	262.22	0.0000	H-1→L+2 73.3%, H-1→L+8 19.7%
10	4.9693	249.50	0.0000	H→L+4 48.3%, H→L+3 25.2%, H→L+9 11.6%, H-4→L 5.1%
11	4.9770	249.11	0.0000	H-8→L 25.1%, H-6→L 24.4%, H-2→L+3 23.6%, H-9→L+1 6.2%,

				H-3→L+2 5.5%
**12**	**5.0722**	**244.44**	**0.5472**	**H-1→L+4 30.5%, H-3→L 15.5%, H-2→L+1 15.2%, H-1→L+3 11.8%, H-1→L+9 9.2%, H→L+2 7.0%**
13	5.1735	239.65	0.0119	H-2→L+2 17.6%, H-6→L+1 17.6%, H-9→L 15.8%, H-3→L+3 14.0%, H-8→L+1 13.9%, H-7→L 13.0%
14	5.1940	238.71	0.0000	H→L+3 31.1%, H→L+4 27.1%, H-4→L 21.7%
15	5.2026	238.31	0.0009	H-3→L+2 27.5%, H-9→L+1 26.6%, H-7→L+1 22.2%, H-6→L 6.5%, H-2→L+3 5.1%
16	5.3073	233.61	0.9057	H-1→L+4 44.1%, H-3→L 13.2%, H→L+2 11.6%, H-2→L+1 11.3%, H-1→L+10 7.3%
17	5.6288	220.27	0.3092	H-1→L+3 47.7%, H-5→L 15.1%, H-4→L+1 9.9%, H→L+2 7.6%, H-2→L+1 6.5%
18	5.7581	215.32	0.0000	H-4→L 23.2%, H→L+11 15.8%, H→L+10 14.2%, H→L+3 10.8%, H→L+15 5.3%
19	5.8496	211.95	0.0000	H-5→L+1 50.3%, H-1→L+8 16.5%, H-7→L+2 10.3%, H-11→L+1 6.5%
20	5.8764	210.99	0.0038	H→L+5 63.4%, H→L+13 14.8%, H→L+7 9.6%

Table S9. Selected excited-state descriptors (Sr, D, t, H, HDI, EDI, and Ecoul for C1 and C3 in the gas phase, cyclohexane, acetonitrile, and water.

			Sr (a.u.)	D (Å)	t (Å)	H (Å)	HDI	EDI	E_{coul} (eV)
C1	Gas	S2	0.66	0.90	-1.31	2.96	8.14	7.23	5.41
	c-C_6H_6	S2	0.77	0.93	-1.50	2.98	7.20	6.70	5.37
	CH_3CN	S2	0.77	0.93	-1.62	2.96	7.20	6.69	5.42
	H_2O	S2	0.77	0.93	-1.63	2.96	7.20	6.69	5.43
C3	Gas	S2	0.31	5.02	3.71	2.78	9.61	5.68	2.71
	c-C_6H_6	S2	0.32	4.87	3.56	2.83	9.86	5.85	2.76
	CH_3CN	S2	0.33	4.71	3.41	2.88	10.78	6.40	2.81
	H_2O	S3	0.33	4.68	3.39	2.89	10.81	6.42	2.82

Note: Sr, D, t, H, HDI, EDI and E_{coul} denote Sr index (integral of Sr function), D index, H index, t index, hole delocalization index, Electron delocalization index and Coulomb attractive energy, respectively.

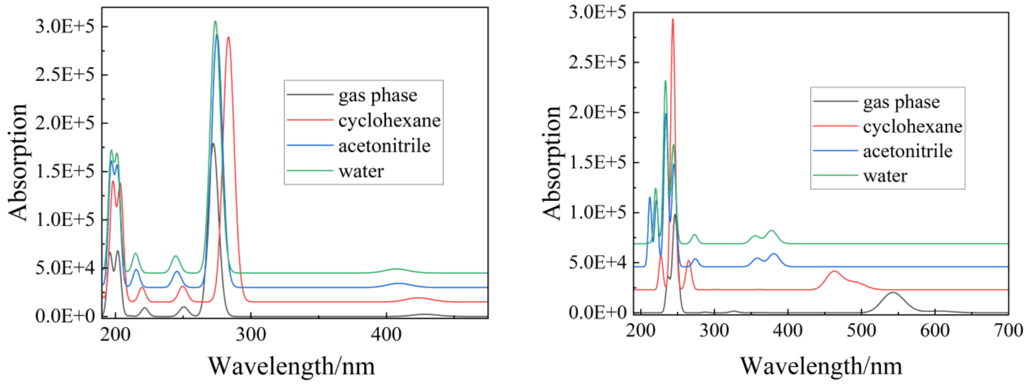

Figure S9. Simulated UV–Vis absorption spectra of compounds C1 (left) and C3 (right) in the gas phase, cyclohexane, acetonitrile, and water (FWHM = 0.15 eV).

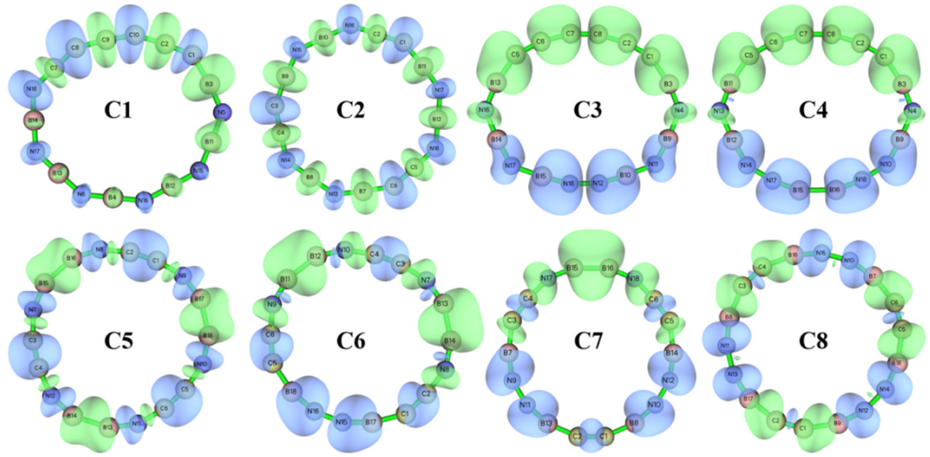

Figure S10. Isosurfaces of the orbital-weighted dual descriptor for compounds C1-C8. (isovalue = 0.001 a.u.).

Blue and green isosurfaces represent negative and positive values, respectively.

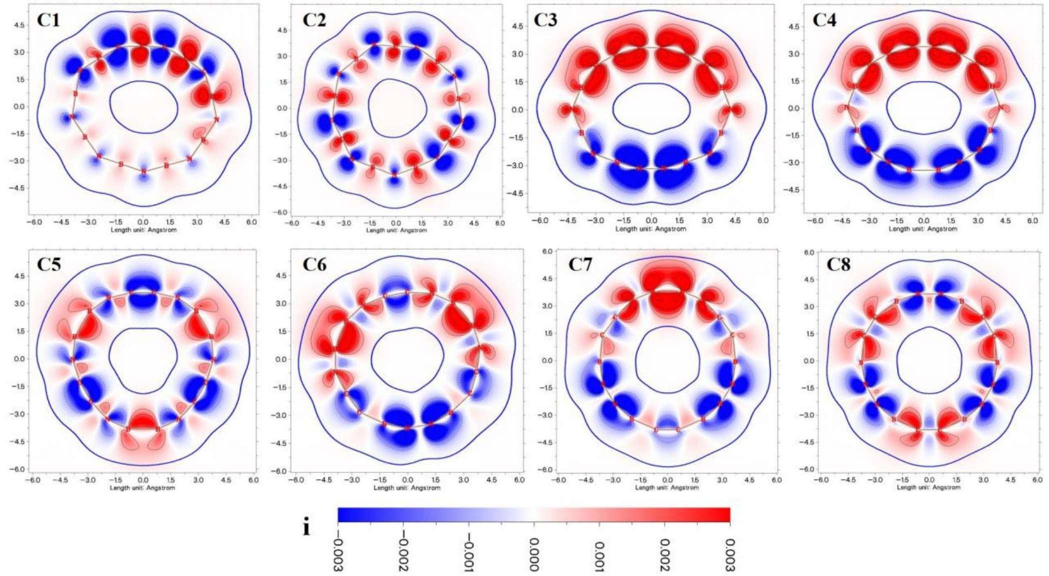

Figure S11. Color-filled maps of the orbital-weighted dual descriptor isosurfaces.
